# The autocrine loop of ALK receptor and ALKAL2 ligand is an actionable target in consensus molecular subtype 1 colon cancer

**DOI:** 10.1186/s13046-022-02309-1

**Published:** 2022-03-29

**Authors:** Martina Mazzeschi, Michela Sgarzi, Donatella Romaniello, Valerio Gelfo, Carola Cavallo, Francesca Ambrosi, Alessandra Morselli, Carmen Miano, Noemi Laprovitera, Cinzia Girone, Manuela Ferracin, Spartaco Santi, Karim Rihawi, Andrea Ardizzoni, Michelangelo Fiorentino, Gabriele D’Uva, Balázs Győrffy, Ruth Palmer, Mattia Lauriola

**Affiliations:** 1 Department of Experimental, Diagnostic and Specialty Medicine (DIMES), University of Bologna, Bologna, Italy; 2 Laboratory of Preclinical Studies for Regenerative Medicine of the Musculoskeletal System (RAMSES), (IRCCS) Istituto Ortopedico Rizzoli, Bologna, Italy; 3 Pathology Unit, Maggiore Hospital, AUSL Bologna, Bologna, Italy; 4 National Laboratory of Molecular Biology and Stem Cell Engineering, National Institute of Biostructures and Biosystems (INBB), Bologna, Italy; 5 Institute of Molecular Genetics, National Research Council of Italy, Bologna, Italy; 6 IRCCS-Institute Orthopaedic Rizzoli, Bologna, Italy; 7 Medical Oncology, IRCSS Azienda-Ospedaliero Universitaria di Bologna, Via Albertoni 15, 40138 Bologna, Italy; 8 Semmelweis University Department of Bioinformatics and 2nd Department Of Pediatrics, Budapest, Hungary; 9 TTK Cancer Biomarker Research Group, Institute of Enzymology, Budapest, Hungary; 10 Department of Medical Biochemistry and Cell Biology, Institute of Biomedicine, Sahlgrenska Academy, University of Gothenburg, Gothenburg, Sweden; 11 Centre for Applied Biomedical Research (CRBA), Bologna University Hospital Authority St. Orsola -Malpighi Polyclinic, Via Belmeloro 8, 40125 Bologna, Italy

**Keywords:** ALK, ALKAL2, CMS1, Colon Cancer therapy, Signalling, AKT, 3D

## Abstract

**Background:**

In the last years, several efforts have been made to classify colorectal cancer (CRC) into well-defined molecular subgroups, representing the intrinsic inter-patient heterogeneity, known as Consensus Molecular Subtypes (CMSs).

**Methods:**

In this work, we performed a meta-analysis of CRC patients stratified into four CMSs. We identified a negative correlation between a high level of anaplastic lymphoma kinase (ALK) expression and relapse-free survival, exclusively in CMS1 subtype. Stemming from this observation, we tested cell lines, patient-derived organoids and mice with potent ALK inhibitors, already approved for clinical use.

**Results:**

ALK interception strongly inhibits cell proliferation already at nanomolar doses, specifically in CMS1 cell lines, while no effect was found in CMS2/3/4 groups. Furthermore, in vivo imaging identified a role for ALK in the dynamic formation of 3D tumor spheroids. Consistently, ALK appeares constitutively phosphorylated in CMS1, and it signals mainly through the AKT axis. Mechanistically, we found that CMS1 cells display several copies of ALKAL2 ligand and ALK-mRNAs, suggesting an autocrine loop mediated by ALKAL2 in the activation of ALK pathway, responsible for the invasive phenotype. Consequently, disruption of ALK axis mediates the pro-apoptotic action of CMS1 cell lines, both in 2D and 3D and enhanced cell-cell adhesion and e-cadherin organization. In agreement with all these findings, the ALK signature encompassing 65 genes statistically associated with worse relapse-free survival in CMS1 subtype. Finally, as a proof of concept, the efficacy of ALK inhibition was demonstrated in both patient-derived organoids and in tumor xenografts in vivo.

**Conclusions:**

Collectively, these findings suggest that ALK targeting may represent an attractive therapy for CRC, and CMS classification may provide a useful tool to identify patients who could benefit from this treatment. These findings offer rationale and pharmacological strategies for the treatment of CMS1 CRC.

**Supplementary Information:**

The online version contains supplementary material available at 10.1186/s13046-022-02309-1.

## Background

Several efforts have been made to stratify colorectal cancer patients into molecularly homogeneous subgroups, in order to unveil prognostic and predictive factors, which may identify specific treatment regimens [1]. To assemble and organize CRC classifications, obtained by different approaches [2–5], Guinney and colleagues developed a unifying association network that successfully identified four consensus molecular subtypes (CMSs), each one with specific biological features [6]. Notably, these transcriptional subtypes are maintained in CRC cell lines [3, 7], thus allowing the selection of a precise in vitro model to recapitulate at the best the investigated patient subtypes.

Anaplastic lymphoma kinase (ALK) is a large, glycosylated receptor tyrosine kinase (RTK) that belongs to the superfamily of the insulin receptors, showing a great similarity to leukocyte receptor tyrosine kinase (LTK, 79% identity) [8, 9]. RTKs undergo ligand-mediated activation, resulting in receptor phosphorylation in response to ligand [10–12]. The small secreted ALKAL proteins (ALKAL1 and ALKAL2), also known as FAM150A/B and augmentor-α/β, have been reported as activating ligands for ALK [13, 14]. Besides, in a recent work, ALKAL1 expression has emerged to positively correlate with CRCs malignancy and bad prognosis [15]. Physiologically ALK is expressed in the nervous system to regulate proliferation, differentiation and survival, and its expression level decreases after birth [16]. ALK is generally poorly expressed in normal adult tissues, making it a highly promising molecular target for cancer therapy [17, 18]. Indeed, ALK is known to be oncogenic in different types of cancer [19] such as Non-Small Cell Lung Cancer (NSCLC) and it has a critical role in neuroblastoma [20], both as a consequence of gene translocation [21], overexpression, point mutations [11, 22] or hyper-phosphorylation of the downstream pathways [23]. ALK gene copy number gain was also detected in both the alveolar (88%) and embryonal (52%) rhabdomyosarcoma subtypes [24]. Of note, the involvement of the ALK full-length receptor and its autocrine activation in colorectal cancer has so far been poorly investigated. CRC patients harboring *ALK* gene amplification or copy number gain display worse prognosis [25, 26], while *ALK* genomic rearrangements, such as translocations or fusion proteins, are rarely identified [27–29].

Here we show that full-length *ALK* expression has a robust predictive ability in the survival of CRC patients belonging to CMS1. Stemming from these findings, we developed in vitro and in vivo molecular assays, probing the inhibition of ALK to investigate the benefit of therapies targeting this pathway. We observed an autocrine activation of ALK signaling by its cognate ligand ALKAL2, in CMS1. These results were coupled with confocal imaging of the 3D spheroids growth and evaluation of the invasive phenotype. Strikingly, ALK inhibition was effective in inhibiting proliferation in CMS1 both in vitro and in vivo in animals, while no effects were detected in the other subtypes. Mechanistically, ALK/ALKAL2 appears to signal mainly through the AKT axis, which was successfully nullified by ALK inhibition, leading to massive apoptotic death. Moreover, ALK signature was associated with survival specifically in the CMS1 dataset, confirming the whole pathway activation in CMS1. Finally, evaluation of patient-derived organoids and an in vivo xenograft mouse model, confirmed that ALK inhibition may represent a novel therapeutic opportunity for CMS1 patients.

## Methods

### Cell culture and transfection

Experiments were performed using several colorectal cancer (CRC) cell lines and patient-derived organoids (*n* = 3), kindly provided by Prof. Livio Trusolino laboratory. LoVo, HCT116, Caco-2, HT29, LS174T were grown in Dulbecco’s Minimal Essential Medium (DMEM) high-glucose, LS1034 and NCI-H508 were grown in Roswell Park Memorial Institute (RPMI) 1640, while SW48 were grown in Leibovitz medium (L-15). Media were supplemented with 10% fetal bovine serum (FBS) and 1% penicillin-streptomycin. Organoids were cultured in 100% Cultrex® RGF BME (R&D Systems, Minneapolis, USA) covered with DMEM/F12 (1% penicillin-streptomycin and 2 mM L-Glutamine) supplemented with B-27 1X, N-2 1X, 1 mM N-acetylcysteine and EGF 10 ng/ml. Cells and organoids were cultured in a humidified 37 °C incubator with 5% CO_2_, except SW48 cell line, which has been kept in dioxide-free conditions as required for L-15 medium maintenance. All cell lines were routinely tested for *Mycoplasma* contamination by PCR. All cell lines were validated from the external services Eurofins Medigenomix, Ebersberg and BMR Genomics srl, Padova, Italy. ALKAL1/2 containing medium was produced in HEK293 cells by Lipofectamine 2000 transfection, according to manufacturer instructions. ALKAL1/2 expressing plasmids have been previously described [14].

### Proliferation and colony forming assays

For AlamarBlue (resazurin) proliferation assays, cells were seeded in full medium in 96- well plates. Quantification of initial time 0 was performed the following day, adding 200 μM of AlamarBlue. Cells were then treated according to the experiment and proliferation was measured again after 96 h. In order to perform the colony forming assays, cells were seeded in full medium in 12-well plates and treated the following day according to the experiment. After 10 days, cells were fixed in 4% PFA and stained with Crystal Violet 0.5%. For both the AlamarBlue and colony forming assays, data were analyzed as previously reported [30]. For the BrdU assay, cells were seeded in full medium on coverslips in a 12-well plate and, after 2 days, treated for 24/48 h according to the experiment set-up. At the end of the treatment, cells were incubated with 1 μg/ml BrdU for 20′ or 30′. Cells were then fixed in 4% PFA and later incubated in 2 N HCl at 37 °C for 15′, causing DNA hydrolysis. After treating with 0.2% Triton and blocking with 1% bovine serum albumin (BSA, Sigma-Aldrich, St. Louis, USA), 3 μg/ml anti-BrdU primary antibody was added O/N. The next day, cells were incubated with secondary antibody and DAPI (Sigma-Aldrich, St. Louis, USA). Images were taken using the Olympus BH-2 CCD microscope. Positive cells were counted using the ImageJ software.

### Apoptosis assays

For TUNEL assay, cells were seeded in full medium and treated for 48 h according to the experiment. After fixation in 4% PFA, cells were permeabilized with 0.2% Triton and treated for 1 h with the TUNEL reaction mixture (In Situ Cell Death Detection Kit, POD, Enzo Biochem, New York, USA), except for the negative control where the enzyme was not added. Lastly, cells were incubated with DAPI and the coverslip was mounted onto the slide. Images were taken using the Olympus BH-2 CCD microscope, detecting fluorescence in the range of green. Positive cells were counted using the ImageJ software. For the Annexin V/Propidium Iodide (PI) detection assay, 1 × 10^6^ untreated and CZB-treated cells were collected and stained with APC Annexin V and PI (BioLegend, San Diego, USA) for 15′. Detection was performed with Cytoflex S Flow Cytometer and data analysis was carried out with the CytExpert software.

### Spheroid assay

For spheroid formation assays, cells were grown in total suspension over a layer of sterile agar diluted to 0.6% in full medium. EGF 10 ng/mL was added in certain cases to promote spheroids formation. Treatments were added simultaneously to seeding. After 10–15 days, pictures of four non-overlapping fields for each well were collected. Spheroids of each picture were counted, and the length of the major and minor axis of each spheroid was measured using ImageJ software. Axis values below 70 A.U. were excluded as not corresponding to mature spheroids, and volume was calculated applying the sphere adapted formula (major axis x minor axis^2^) /2.

### Confocal microscopy and spheroids clearing

Confocal microscopy was used to investigate the inner structure of spheroids and potential phenotypical changes in response to drugs treatment. Spheroids from 3D culture were collected, fixed and stained according to the procedure previously described [31]. See the list of reagents in Supplementary Information for additional details about fluorescent dyes. When necessary, spheroids clearing was performed prior to staining and microscope observation. For this purpose, samples were incubated in the X-CLARITY Hydrogel- Initiator solution (Logos Biosystems, Inc. Anyang-Si, Gyunggi-Do, South Korea) according to manufacturer’s instructions. Samples were polymerized with the X- Clarity™ Polymerization System (Logos Biosystems) for 3 h with a vacuum of 90 kPa and a temperature of 37 °C. After polymerization, spheroids were gently shaken for 1′, rinsed several times with PBS and stored at 4 °C. Subsequently, samples-hydrogel hybrids were immersed in an Electrophoretic Tissue Clearing Solution (Logos Biosystems) and placed for 8 h in the X-Clarity™ Tissue Clearing System (Logos Biosystems) with the following settings: current 0.8 A, temperature 37 °C, pump speed 30 rpm. After several washes in PBS, spheroids were then incubated with 4% BSA (Sigma-Aldrich, St. Louis, USA) to avoid unspecific bindings prior to incubation with primary and secondary antibodies. The complete list of the used antibodies can be found in the Supplementary Information section. Samples were then mounted with a mixture of X- CLARITY mounting solution (Logos Biosystems) and 1,4-Diazabicyclooctane (DABCO) (Sigma-Aldrich). Confocal imaging from non-clarified samples was performed with a Nikon A1-R confocal laser scanning microscope, equipped with a 20× 0.7 NA objective and with 405 and 561 nm laser lines to excite DAPI and red fluorescence signals. Fluorescent images from clarified spheroids were visualized and imaged under FV300 fluorescent confocal microscope (Olympus, Shinjuku, Japan) equipped with a 30x (silicon immersion, 1.05 NA) and 60× (silicon immersion, 1.30 NA) super apochromat objectives and with 405, 488, 561 and 647 nm laser lines. Z-stacks were collected at optical resolution of 210 nm/pixel, stored at 12-bit with 4096 different gray levels, pinhole diameter set to 1 Airy unit and z-step size to 1 μm. The data acquisition parameters were setup in fixed manner, such as laser power, gain in amplifier and offset level. Confocal images were processed using Richardson- Lucy *deconvolution* algorithm. Volume view with 3D rendering was carried out using the NIS Elements Advanced Research software (Nikon, Shinagawa, Japan).

### Incucyte® 3d single tumor spheroid assay

Spheroids growth was followed in time-lapse by means of IncuCyte S3® Live-Cell Analysis system (Essen Bioscience, Ann Arbor, USA). For live imaging assays, cells were seeded in 96-wells plates over a layer of 0.6% agar as performed for spheroid assays. Fluorescent reagents for detection of cytotoxicity and apoptosis, designed specifically for IncuCyte S3® experiments by Essen Bioscience, were added at the time of seeding, according to manufacturer’s instructions. More details about the dyes for live-cell experiments can be found in the Fluorescent Probes List in Supplementary Information section. Analyses were performed using IncuCyte S3® Software (v2018C).

### Gelatin degradation assay

Gelatin degradation assay was performed by coating glass coverslips with Oregon green-488 conjugated gelatin from pig skin (Invitrogen™, Waltham, USA) diluted to a final concentration of 0.2 mg/mL in PBS + sucrose 2%. After coating solidification, gelatin was treated for 15′ on ice with cold glutaraldehyde 0.5% in PBS and then with NaBH_4_ 5 mg/mL in PBS for 3′ at room temperature. Coated coverslips were stored at + 4 °C in PBS + P/S 1:50 protected from light until seeding day. 50 k SW48 cells were then seeded and incubated for 24 h to allow cells to settle. For ALKAL1/2 treatment, cells were incubated for 6 h with conditioned medium from HEK293 transfection with ALKAL1/2 plasmids. The coverslips were then fixed with PFA 4% for 15′, blocked for 30′ with BSA3% + 0.1 Triton X-100 and finally stained with Phalloidin-TRITC (Sigma-Aldrich, St. Louis, USA) and DAPI diluted in BSA 0.3% + 0.1 Triton X-100 for 30′. Imaging was performed using the Olympus BH-2 CCD microscope. Images were analyzed using ImageJ software and degradation was measured in the green channel in terms of area of degraded gelatin (without green fluorescence) normalized on nuclei number (DAPI).

### Western blot

Starved and treated cells were lysed using RIPA buffer supplemented with a protease inhibitor cocktail (P8340, Sigma-Aldrich) and 1 mM Na_3_VO_4_ (Santa-Cruz Biotechnology, Dallas, USA). Protein concentration was determined by DC Protein Assay (Bio-Rad, Hercules, USA), using bovine serum albumin as the standard. Protein samples were separated by SDS-PAGE and then transferred to nitrocellulose membranes (Amersham™ Protran™ Premium, GE Healthcare, USA). Membranes were blocked for 1 h using 5% BSA (Sigma-Aldrich) in TBS-T (0.1% Tween-20) and incubated O/N (4 °C) with primary antibodies diluted in the blocking solution. Finally, membranes were incubated with horseradish peroxidase-coniugated secondary antibodies against mouse or rabbit for 1 h and protein presence was detected by chemiluminescent reaction (Amersham™ ECL™ Detection Reagents). Bands quantification was performed using Image Lab software. Details about the antibodies employed for proteins detection are reported in Supplementary Information.

### qRT-PCR and droplet digital PCR (ddPCR)

Total RNA was extracted from starved cells using QIAzol® Lysis Reagent (QIAGEN, Venlo, Netherlands) according to manufacturer’s instructions. qRT-PCR analyses were then performed on cDNA using the SsoFast EvaGreen Supermix (Bio-Rad, Hercules, USA). A complete list of the primers employed is reported in the Supplementary Information section. Gene expression data from qRT-PCRs were determined as ΔCq (Quantification Cycle) normalized on the expression of β_2_-microglobulin (B2M) housekeeping gene.

Droplet Digital PCR (ddPCR) was performed using QX200 ddPCR EvaGreen SuperMix (Bio-Rad, Hercules, CA, USA, cat. no. 1864034) and QX200 Droplet Digital PCR System (Bio-Rad). Briefly, for each target gene a PCR mixture containing 10 μL of 2X QX200 ddPCR EvaGreen SuperMix, primers at final concentration of 250 nM, a variable volume input of cDNA in a final volume of 20 μL. Specifically, for the evaluation of ALK and ALKAL1 expression 100 ng of cDNA were used in the reaction mixture, for each sample, while for B2M and ALKAL2 1 ng. All the experiments included a no template control (NTC). Droplet generation was performed as detailed in [32]. The cycling protocol was 95 °C for 5 min, then 40 cycles of 96 °C for 30 s and 58 °C for 1 min and three final steps at 4 °C for 5 min, 90 °C for 5 min and 4 °C infinite hold. The ramping rate between these steps was set at 2 °C/second. Data analysis was performed using the software Quantasoft v.1.5.38 (Bio-Rad) and the positive droplets were manually selected using the lasso tool. The final concentration of each target gene was obtained in all the analyzed samples, expressed in copies/μL. This concentration was normalized as copy number in 100 ng of cDNA and corrected for B2M expression as reference gene.

### Immunohistochemistry

Routine immunohistochemical staining for ALK in xenograft tumors and LoVo spheroids was performed on a Benchmark-Ultra automated system (Ventana-Roche, Tucson, USA) using pre-diluted antibodies. All specimens have been formalin-fixed and paraffin-embedded (FFPE). The following antibodies were used: ALK D5F3 antibody (Cell Signaling, Danvers, USA), E-cadherin clone 36 mAb (Ventana-Roche, Tucson, USA), Vimentin clone V6 mAb (Ventana-Roche, Tucson, USA). The OptiView DAB IHC Detection Kit was used for detection.

### Animal experiments

Athymic Nude Foxn1nu 6-weeks old female mice, obtained from Envigo (Indianapolis, USA), were injected subcutaneously in the right flank with SW48 cell lines (1X10^6^ per mouse) and randomized into two groups. Crizotinib was purchased from MedChem Express (New Jersey, USA) and administered daily in mice though oral gavage. Tumor width (W) and length (L) were evaluated using a caliper twice a week and Tumor volume (V) was calculated according to the following formula: V = 3.14 × (W^2^ × L)/6. Measurements of the body weight was acquired once per week. Mice were euthanized when tumor size reached 1500 mm^3^.

### Statistics

The statistical analyses were performed by using Prism version 6 (GraphPad Software, Inc). Both T-test and one-way ANOVA were used to test significance of the assays. The details of the applied statistical test are reported in the figure legends. * Pvalue < 0.05; ** Pvalue < 0.01; *** Pvalue < 0.001; **** Pvalue < 0.0001.

## Results

### *ALK* is predictive of survival in consensus molecular subtype 1 CRC patients

**Fig. 1:**
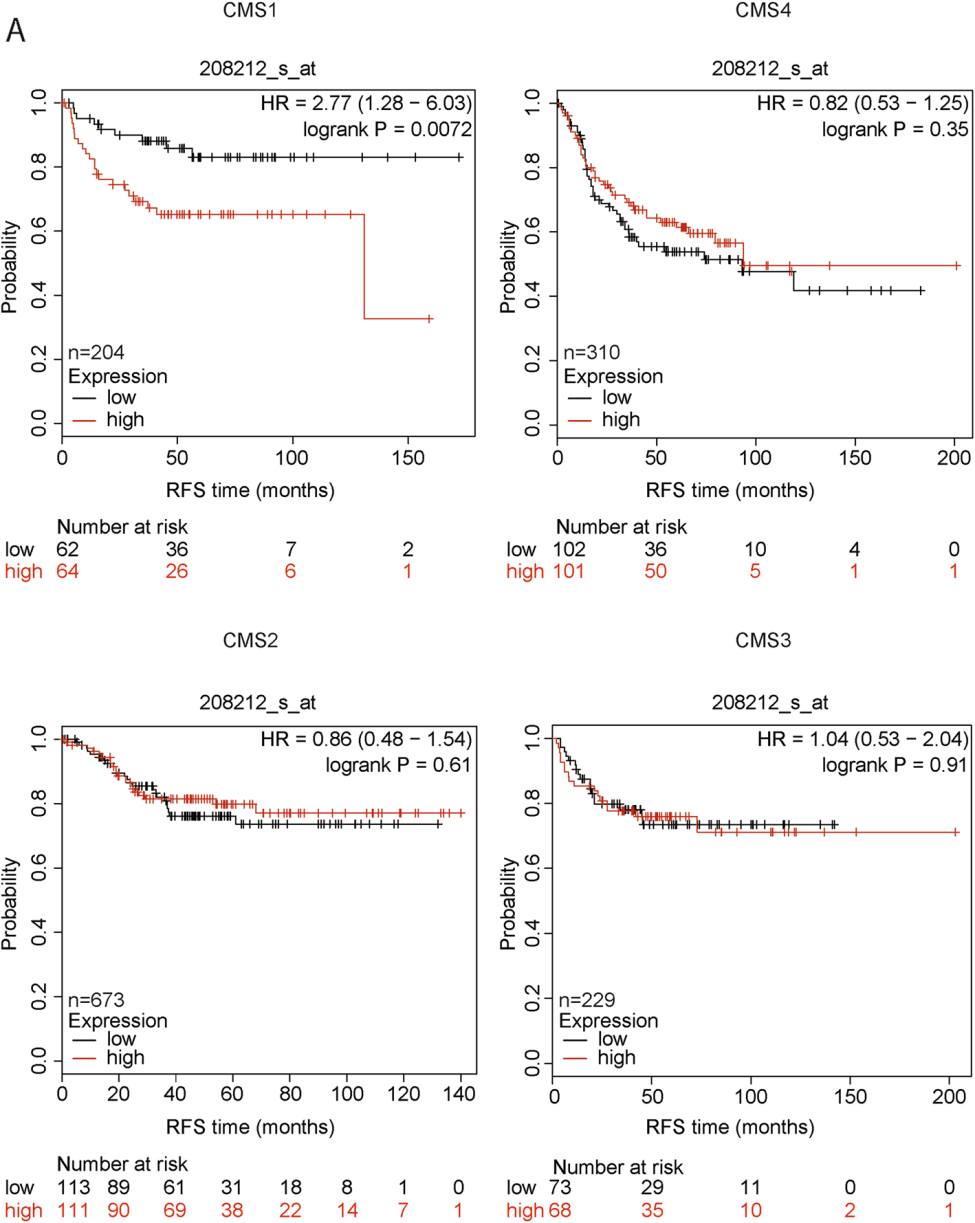
ALK is predictive of survival in consensus molecular subtype 1. Kaplan-Meier analysis of a cohort of 1700 patients stratified by CRC classification and *ALK* gene expression (probe 208212_s_at). The red line represents high level of expression, while the black one is representative of low levels. For each patient, the relapse-free survival (RFS) probability is reported over a period of maximum 200 months

Various colorectal cancer patients display a different set of genomic alterations that lead to a diverse response to therapies. To investigate ALK involvement in colorectal cancer, we started from the analysis of a dataset of CRC patients, stratified for consensus molecular subtypes: CMS1, CMS2, CMS3 and CMS4 [33]. Patients were tested for the amount (high/low) of mRNA expression of ALK (probe 208212_s_at). The relapse-free survival (RFS) probability of these subjects was calculated throughout 200 months (about 16 years). After trichotomization of the dataset, in each molecular subtype separately, the median group was excluded and the groups with high and low expression of ALK mRNA were compared. High levels of ALK were predictive of poor relapse free-survival specifically in CMS1, with an HR of 2.77 and a *P* value of 0.0072 (Fig. 1). Indeed, most of these patients died within 50 months, suggesting that ALK plays a significant prognostic role in CMS1. This trend was specific for CMS1, as CMS2, 3 and 4 subgroups displayed no survival predictive ability for ALK (Fig. 1). CMS1 is characterized by microsatellite instability, and it is linked to hypermutation and hypermethylation (CIMP). It represents 15% of CRCs, which are triggered by alterations in the DNA mismatch-repair system, inducing microsatellite instability (MSI) and a high mutation rate [34]. Next, we searched for a suitable in vitro model recapitulating the features of CMS1 subtype. In the literature, some reports classify CRC cell lines according to the CMS, following the same classification system established for the patients. We employed the classification from Berg and colleagues [35], that assigned each cell line to its specific CMS, corresponding to the patients’ classification. We selected LoVo and SW48 cells as representative models for CMS1 (where ALK is predictive of poor survival), LS1034 and NCI-H508 for CMS2, HT29 and LS174T for CMS3, HCT116 and Caco-2 for CMS4. First, we confirmed the lack of ALK aberrant translocations in these cell lines by FISH staining (Supplementary Fig. 1).

### Antitumorigenic action of ALK axis inhibition is specific for CMS1

**Fig. 2:**
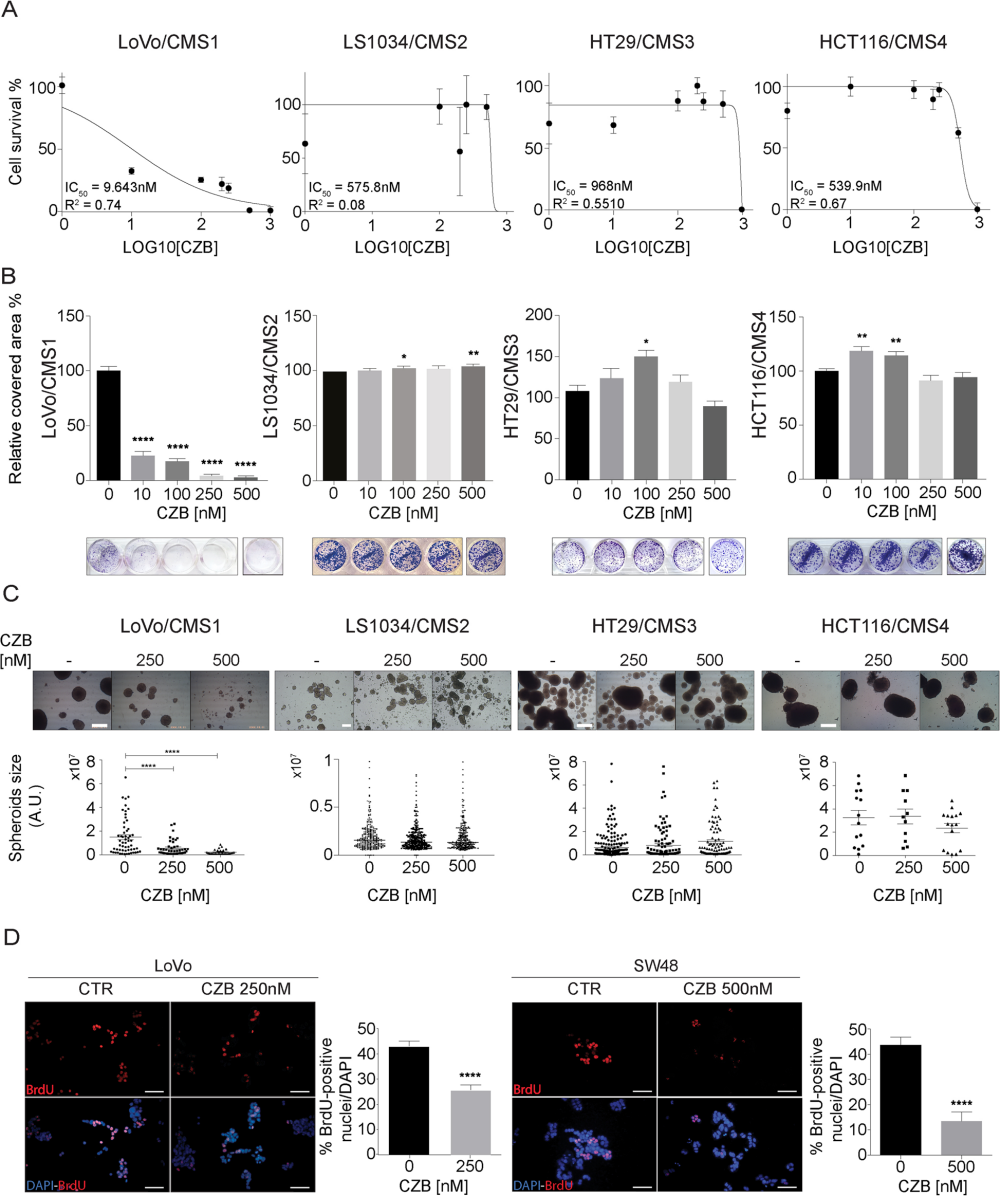
ALK inhibition by CZB reduces proliferation of CMS1 cell lines. **A** Cell survival assessment upon CZB addition by Alamar Blue assays. A panel of four CRC cell lines belonging to different CMSs (LoVo-CMS1, LS1034-CMS2, HT29-CMS3, HCT116-CMS4) was tested with increasing doses of CZB. Data are reported as % of cell proliferation in relation to controls ± SD. IC50 values are shown for each cell line. **B** Colony forming assays of cell lines in (**A**) treated with increasing concentrations of CZB. Representative images of treated and untreated wells are shown. Cell viability data are reported as % of wells covered area in relation to controls ± SD. Statistics were calculated by one-way ANOVA test. **C** Spheroid-forming assays of cell lines in (**A**) treated with CZB 250 nM and 500 nM. Statistic was calculated by one-way ANOVA. Scale bars: LoVo, HT29, HCT116: 250 μm. LS1034: 200 μm. **D** BrdU incorporation assays of LoVo and SW48 upon CZB treatment. Representative images show BrdU-positive nuclei in red, while total DAPI-stained total nuclei are visible in blue. Quantifications of BrdU-positive nuclei in relation to total nuclei ± SEM is shown. Two tailed unpaired T-test was applied. Scale bar: 25 μm

We employed two different ALK inhibitors, crizotinib (CZB) and alectinib (ALC), which are currently used for the treatment of ALK-positive NSCLC patients. To experimentally examine the patient data-driven hypothesis of ALK pathway involvement in CMS1 colon cancer subtype, we treated CMS1 (LoVo and SW48), CMS2 (LS1034 and NCIH508), CMS3 (HT29 and LS174T) and CMS4 (HCT116 and Caco-2) cell lines with increasing concentrations of CZB or ALC. ALK inhibition strongly affects LoVo and SW48 cells proliferation, while almost no effect was recorded in the other cell lines (Fig. 2A and Supplementary Fig. 2A and 3D). These results were consistent with the colony forming ability assay, that measures the percentage of cells retaining the capacity of producing several progenies, detected as colonies (Fig. 2B and Supplementary Fig. 2B and 3A). To further validate these results, we tested ALK inhibition by CZB and ALC on a system closer to tumors in vivo. Cells were seeded in adhesion lacking conditions, forced to grow in suspension, in three-dimensional structures, known as spheroids, as previously reported [30, 31]. Multicellular spheroids reproduce the 3D architecture, cell-cell interactions as well as oxygen and proliferation gradients observed in tumors [36]. Spheroids were left to grow for approximately 15 days and the size of each spheroid under the vehicle or CZB/ALC treatments was measured. In these conditions, we found that ALK inhibition strongly affects LoVo and SW48 growth (Fig. 2C and Supplementary Fig. 2C and 3B). On the other hand HCT116, HT-29, LS1034 and NCI-H508 cells grow completely undisturbed, even at high doses of drugs (Fig. 2C and Supplementary Fig. 2E and 3E), suggesting that ALK inhibition is not active on CMS2/3 and CMS4 cells, while CMS1 cells are highly sensitive. Next, we applied a soft agar assay, which represents a surrogate assay for tumorigenesis. It assesses the impact of inhibitors on cancer cell 3D colony formation and is frequently the first experimental validation that compounds, already known to be active in 2D, must possess to progress through the drug discovery pipeline. Under these growing conditions, ALK inhibition of growth resulted again impressive in CMS1 cell lines, while no effect was detected in CMS4 cells (Supplementary Fig. 2D and 3C). To sum up, at doses of 250 nM, very few colonies and spheroids were detected with LoVo and SW48 cells (CMS1), while minimal effect was observed in cells belonging to the other subtypes. Consistently, in CMS1 cell lines, the S phase entry evaluation by BrdU incorporation was strongly suppressed (Fig. 2D). Altogether, these data suggest that CMS1 cell lines depend on ALK pathway for proliferation and survival, while CMS2/3 and 4 cells are less vulnerable to its inhibition.

**Fig. 3:**
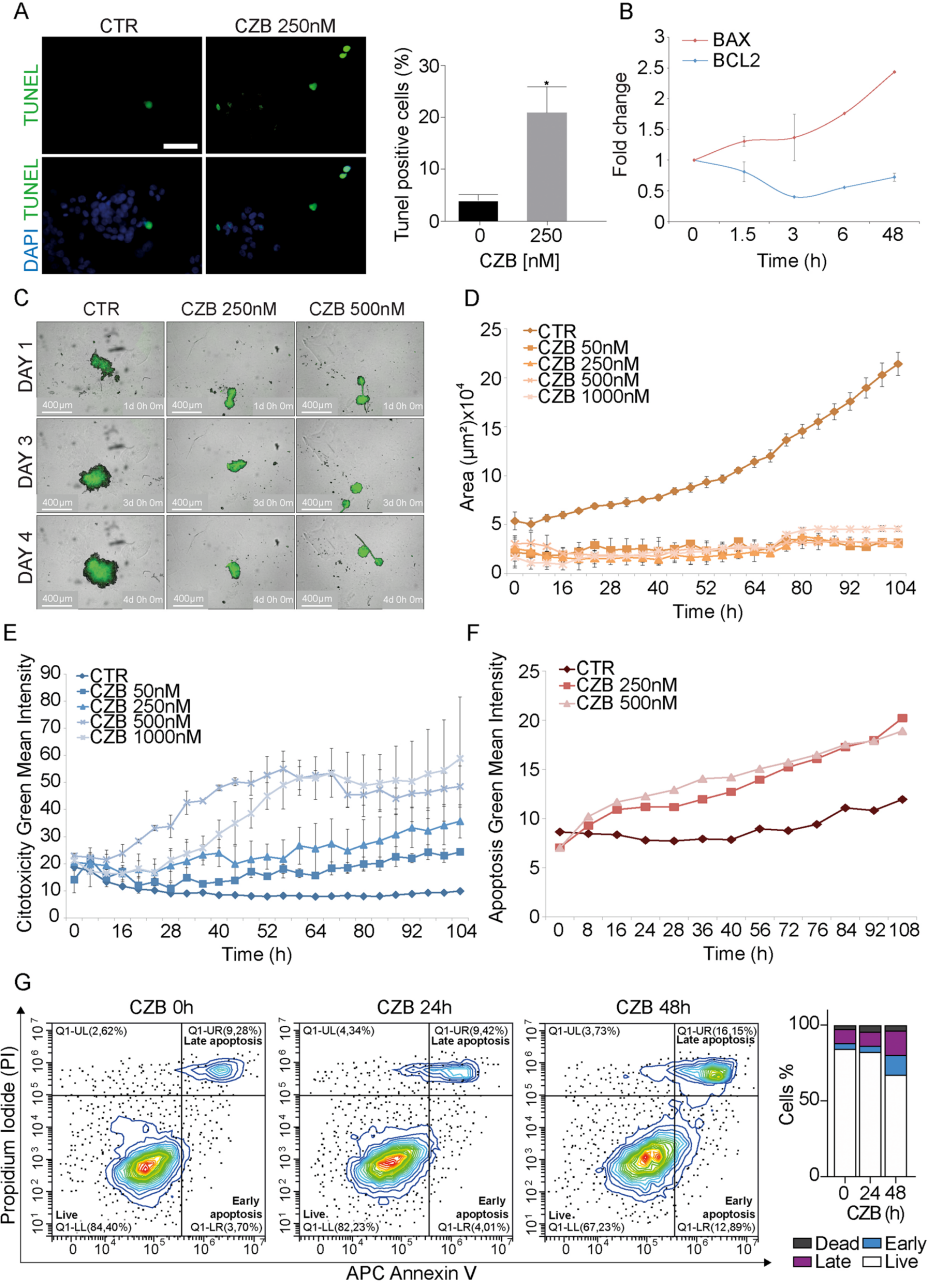
ALK interception triggers apoptotic cell death in CMS1 subtype. **A** Representative images (left) and quantification (right) of double-strand breaks (DSBs) assessment by TUNEL assay in LoVo cells on CZB treatment. TUNEL positive nuclei are shown in green channel, while total nuclei are visualized with DAPI in blue. The number of TUNEL positive nuclei was measured for CTR and CZB conditions, on 20 and 36 not overlapping fields, by analyzing a total of 884 and 972 cells, respectively. Two tailed unpaired T- test was applied for statistical analysis. Scale Bar: 50 μm. **B** Determination of apoptotic markers expression by qPCR analysis performed on LoVo cells RNA, treated with CZB over a 48 h time course. **C** Representative images of LoVo spheroids captured with the IncuCyte® Live-Cell Analysis System. **D**, **E** Quantification of the largest brightfield object area (μm^2^) and largest brightfield object green fluorescence mean intensity (GCU) from a live-cell imaging assay performed on LoVo spheroids cultured as in (**C**) and treated with four different CZB doses (50 nM, 250 nM, 500 nM, 1000 nM). Spheroids were labelled with IncuCyte® Cytotox Green Reagent. Error bars refers to standard error between different replicates. **F** Quantification of LoVo spheroids labelled with IncuCyte® Caspase-3/7 Green Reagent to detect apoptotic cells. **G** FACS analysis of LoVo/CMS1 cell line assessing the percentage of cells undergoing apoptosis in full medium-growing conditions and after CZB treatment (24 h or 48 h), by means of Annexin V detection. Propidium Iodide (PI) was added to help differentiating between apoptotic and necrotic cells. Quantification is provided on the right

Previous studies reported a role for ALK in the inhibition of apoptosis in uterine carcinosarcoma [37]. Thus, we evaluated apoptosis at single cell level, by the terminal deoxynucleotidyl transferase (TdT)-mediated deoxyuridine triphosphate (dUTP)-biotin nick end-labelling (TUNEL) method. ALK inhibition during a timeframe of 48 h was sufficient to increase TUNEL positivity in CMS1 cells (Fig. 3A). Consistently, the mRNA levels of the antiapoptotic marker BCL2 decreased, while the pro-apoptotic BAX steadily increased, reaching the maximum at 48 h (Fig. 3B). Time lapse imaging using a custom autofocusing method, capturing bright-field and fluorescence images every 6 h for 4 days, confirmed the apoptosis phenotype. Indeed, control CMS1 cells formed large aggregates within the first 3 days, with a diameter of approximately 400 μm (Fig. 3C). The IncuCyte® bright-field image analysis algorithm computed the area and average size of the spheroid over time, non-invasively. ALK inhibition dramatically affected spheroids growth, as revealed by the reduction in the calculated average bright-field area (Fig. 3D). Using green fluorescent label reporters for cytotoxicity, we assessed the ALK inhibition effect on living cells. Indeed, CZB displayed a robust dose-dependent response in inducing cytotoxicity (Fig. 3E), that was strongly triggered by apoptotic pathways, as detected by Caspase-3/7 Green labelling (Fig. 3F). Time-lapse video data are available as Additional files 10, 11, 12, 13, 14 and 15. Finally, fluorescently labeled Annexin V (Annexin V (APC)) and propidium iodide (PI) staining in CMS1 cells LoVo and SW48, confirmed the apoptotic death induced by CZB (Fig. 3G and Supplementary Fig. 4A-B). Annexin V+/PI− LoVo early apoptotic cells and Annexin V+/PI+ late apoptotic cells were shown to increase overtime under CZB treatment, with a total of 28% of apoptotic cells after 48 h (Fig. 3G). Similar results were found for SW48, which recorded a total of 45% of apoptotic cells after 48 h of CZB treatment (Supplementary Fig. 4A-B).

In conclusion, the set of in vitro assays performed both in monolayer and in 3D, confirmed the enhanced antitumorigenic potential of ALK axis inhibition achieved by apoptotic death, specifically in CMS1.

### ALKAL2 stimulates ALK downstream signaling in CMS1

**Fig. 4:**
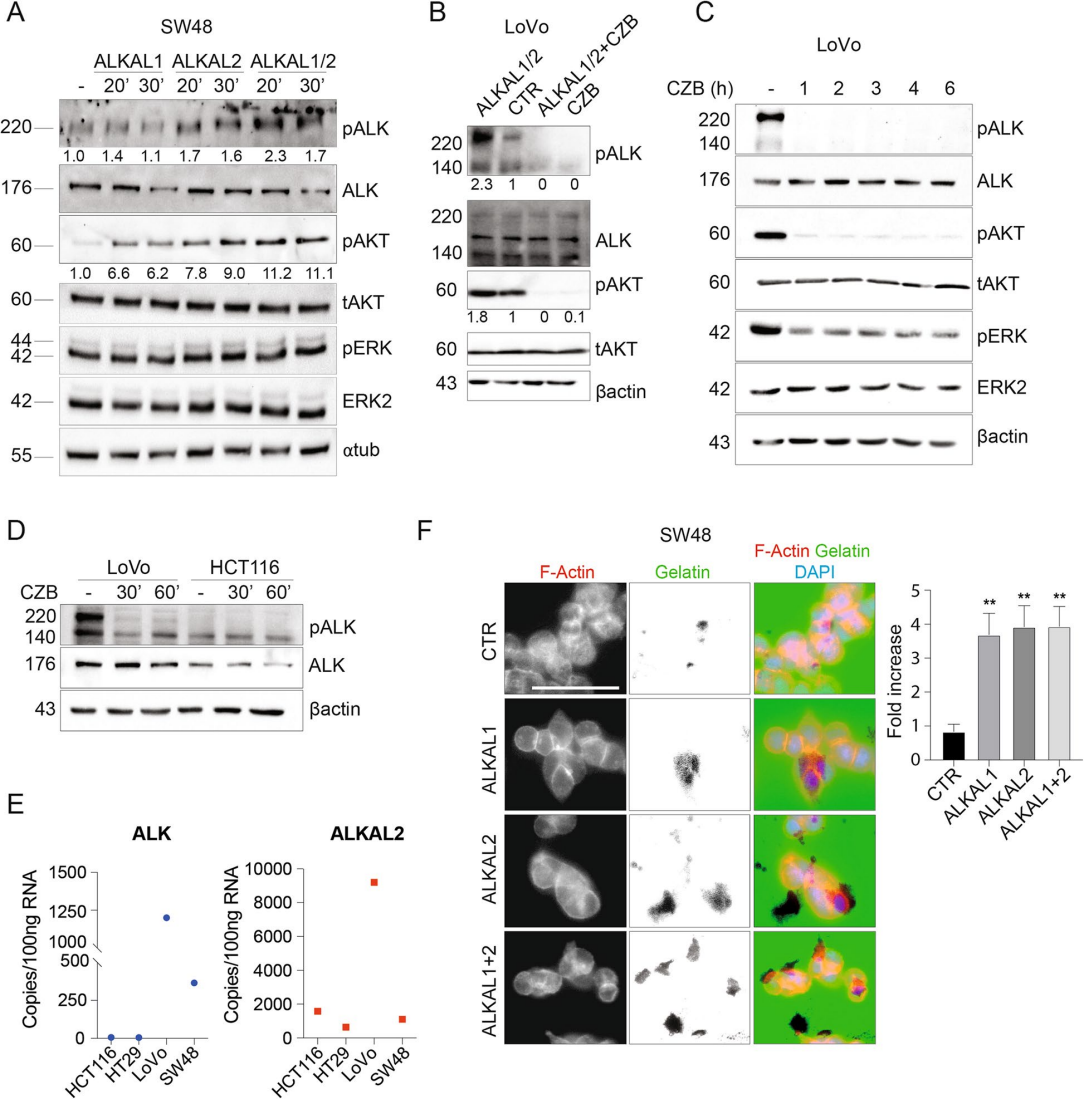
ALK downstream signalling pathway is enhanced upon ALKAL2 activation and dampened upon CZB treatment in cms1 cells. **A** ALK downstream signaling activity in SW48 (CMS1) cells treated with conditioned medium containing ALKAL1, ALKAL2 or their combination for either 20′ or 30′. pALK/ALK and pAKT/AKT quantification is provided. ⍺-tubulin was used as loading control. **B** Investigation of ALK and its downstream pathway activation upon stimulation with ALKAL1/2 containing medium for 30′ in LoVo cells, with or without 30′ CZB pre-treatment. Quantification of pALK and pAKT is shown. **C** Evaluation of ALK, pERK and pAKT inhibition in starved LoVo (CMS1) cells, following treatment with CZB 1 μM over time, from 1 to 6 h. β-actin along with total AKT and ERK2 were used as loading control. **D** ALK expression and activation assessment in LoVo (CMS1) and HCT116 (CMS4) cells in basal conditions and upon 30′ and 60′ CZB 1 μM treatment. β-actin was used as loading control. **E** Droplet-digital PCR performed on RNA extracted from cells belonging to CMS4 (HCT116), CMS3 (HT29) and CMS1 (LoVo, SW48 xenografts). The expression of ALK and ALKAL2 is reported as copy number in 100 ng of RNA, normalized for B2M expression. **F** Gelatin-degradation assay performed on SW48 treated with ALKAL1/2-containing medium. Cells were stained with Phalloidin-TRITC (F-Actin) and DAPI (nuclei) prior to visualization. Invasion was quantified in terms of area of degraded gelatin (black spots visible in the green channel) normalized over the nuclei count in the same field. At least 7 fields were analyzed for each condition. Number of cells analyzed, CTR: *n* = 843, ALKAL1: *n* = 569, ALKAL2: *n* = 268 and ALKL1 + 2: *n* = 491. Results are reported as fold increase related to control. One-way ANOVA test was applied. Scale bar: 50 μm

In terms of signaling output, ALK affects cell growth by activation of multiple signaling pathways, including MAPK and PI3K-AKT [38]. First, we tested the ALKAL1 and ALKAL2 ligands that bind the extracellular domain of ALK leading to a robust activation of the pathway [13, 14]. Stimulation with ALKAL1/2 ligands, alone and in combination, resulted in a strong activation of the ALK-Y1604 and AKT phosphorylation, detected already after 20 and 30 min of treatment (Fig. 4A). Notably, only a mild or no effect was recorded on the ERK axis (Fig. 4A). Next, we confirmed that the sensitivity to CZB was maintained under ALKAL1/2 stimulation, which mediated the activation of ALK and AKT (Fig. 4B). Interestingly, using the ALK D5F3 antibody, we were able to detect two specific bands corresponding to ALK receptor. Indeed, ALK WT undergoes extracellular domain (ECD) cleavage by matrix metalloproteinase 9. The ALK protein is expressed as a 220-kDa form, representing the heavily glycosylated, full-length membrane receptor and as a shorter 140-kDa product, resulting from cleavage or shedding of the N-terminal ECD [21, 39, 40]. In order to gain a better insight into CZB activity, we performed a 6 h time-course on the LoVo-CMS1 cell line (Fig. 4C). The protein analysis unveiled a strong reduction of ALK phosphorylation, already after 1 h of treatment, along with a complete dampening of the AKT downstream pathway. A milder effect was observed on ERK signaling, in line with the results from ALK activation by ALKAL2 that seems to signal mainly thought the AKT axis (Fig. 4A). The same effect on pALK inhibition was reproduced using alectinib both in LoVo spheroids and in the SW48 cell line (CMS1) (Supplementary Fig. 5A-B). Surprisingly, the CZB time-course performed on the HCT116-CMS4 cell line, revealed a substantial increase in both pAKT and pERK levels soon after CZB addition, as reported in Supplementary Fig. 5C. These data may explain the paradoxical mild positive effect on the proliferation of HCT116, that we observed with CZB (Fig. 2B).

Moreover, we tested whether the amount of ALK, both at protein and mRNAs level, was responsible for the different sensitivity to ALK inhibition detected in vitro. Of note, CMS1 cells display very high level of basal ALK activation (ALK-Y1604) along with total ALK (both the 220 and the 140 cleaved-band) (Fig. 4D and Supplementary Fig. 6A). ALK presence in CMS1 cells’ membranes was further confirmed by FACS analysis (Supplementary Fig. 6B). We finally evaluated the ALK mRNA copy number, by Droplet Digital Polymerase Chain Reaction ddPCR. This relatively new technique allows the absolute quantification of target mRNAs copies without the need for standard curves, allowing for significant enhanced sensitivity of standard real-time PCR. We tested CMS1, CMS3 and 4 cell lines. The analysis confirmed the highest abundance of ALK mRNA levels in the LoVo cell line and in SW48-derived xenografts, both belonging to CMS1, with copy number RNA ranging from 1200 to 365 respectively. The CMS3 and 4 instead were negative for ALK mRNA detection (Fig. 4E). Notably, also ALKAL2 ligand-mRNA copy number was impressively high in the LoVo cell line, suggesting an autocrine loop mediated by ALKAL2 in the activation of ALK pathway, specifically in this cell line (Fig. 4E). By contrast, the expression of ALKAL1 was almost completely absent in all the cell lines we tested, both CMS1, CMS3 and 4 (Supplementary Fig. 5D). These data are in line with the reported upregulation of ALK ligand in a collection of colon cancer cells [15].

Given the importance that ALK activation holds in CMS1 cells, we asked whether the aggressiveness of cells, measured in terms of capability to invade, may be influenced by ligands administration. Thus, we seeded SW48 cells on a layer of fluorescent gelatin, and performed a 6 h stimulation with ALKAL1/2 supplemented medium. Strikingly, cells displayed an enhanced ability to degrade the substrate after exposure to ALKAL1/2 ligands, as detected by analyzing the dark spots visible in the green channel (quantification is provided on the right) (Fig. 4F).

To sum up, these data support a CRC tissue specificity for ALKAL2, confirmed by the ability of ALKAL2 ligand to strongly prompt ALK phosphorylation, compared to ALKAL1. The CZB sensitivity was not modified by the ALKAL2 activation of ALK, while the invasive potential was enhanced.

### ALK inhibition leads to increased adhesion and cell-cell interaction

**Fig. 5:**
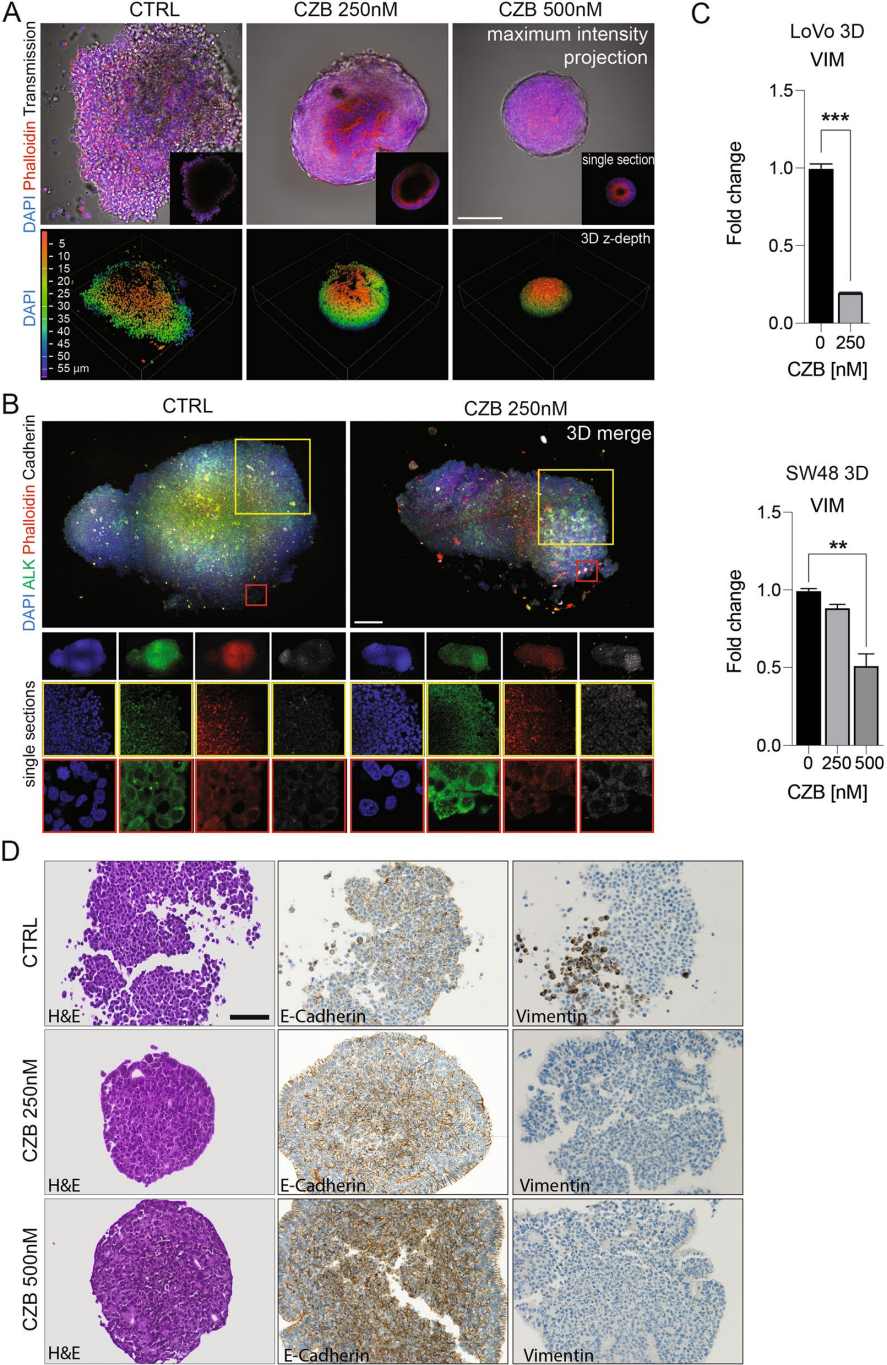
ALK inhibition drives cell-cell adhesion enhancement in CMS1 cells cultured in 3D. **A** Confocal analysis of fixed LoVo cultured in 3D. DAPI (blue) was used to stain nuclei, while Phalloidin was used to stain actin cytoskeleton (red). The upper panel shows maximum intensity projection and single sections (insets) from control and treated spheroids. In the lower panel a volume view with 3D rendering is shown. **B** Confocal analysis of clarified LoVo spheroids, stained with anti-ALK (green) and anti-E-Cadherin (grey) antibodies. Phalloidin (red) and DAPI (blue) were used to stain actin and nuclei respectively. Both 3D merges and single sections at different magnifications for each channel are shown. **C** Determination of vimentin (VIM) expression by qPCR in LoVo and SW48 cells cultured in 3D settings and treated with CZB for 8–10 days. Data are reported in relation to untreated control. Unpaired T- test and One-Way ANOVA were applied for statistical analysis. **D** IHC of LoVo spheroids collected after 15 days of growth, in full medium or under CZB treatment (250 nM or 500 nM). All specimens were formalin-fixed and paraffin-embedded. In the left column Hematoxylin and Eosin staining is provided (scale bar: 100 μm). In the central and right columns, we display respectively E-cadherin and Vimentin IHC (200X magnification)

Confocal microscopy analysis of CMS1 spheroids was employed to analyze the role of ALK in the 3D cell architecture. In line with the previously reported association of ALK with the EMT phenotype [41], under ALK inhibition, the surface organization of the spheroids displayed cell-cell tightness and increased nuclear density, with a smaller lumen cavity (Fig. 5A). Providing clear insights on the internal morphology of large spheroids or in general a thick specimen is challenging, mainly due to the poor penetration of antibodies during the staining or the hampered light penetration. Thus, to further address the evaluation of the enhanced cellular adhesion, we employed X-Clarity™ Tissue Clearing System, which is able to create a stable and optically transparent tissue-hydrogel hybrid [42, 43]. Both ALK and E-cadherin staining were analyzed (Fig. 5B and video as Additional files 16 and 17), indicating that ALK inhibition affects the physical confinement of the nuclear geometry, by dramatically increasing cellular density on the outmost layer of the spheroids, while the cell-cell adhesion is supported by an overall enhanced E-cadherin lateral membrane localization (Fig. 5B and Additional files 16 and 17). To better evaluate the 3D structure organization, we performed immunohistochemistry for the E-cadherin and the mesenchymal marker, vimentin. The 3 μm sections of 3D spheroids show a strong and heterogenous vimentin localization in untreated cells, with a clear subpopulation of positive cells, and an overall mild E-cadherin staining (Fig. 5D). Notably, the CZB treatment strongly enhances cell-cell adhesion by increasing E-cadherin staining, while decreasing the vimentin positive cells. Of note, the outmost layer of treated spheroids shows a columnar epithelium, typical of a differentiated gastrointestinal tract (Fig. 5D). Under CZB treatment, the vimentin drop down was validated also at mRNA levels (Fig. 5C). Interestingly, ALK appears regulated under CZB treatment, with increased membrane/cytosolic expression and localization (Fig. 5B). Further experiments, in 3D spheroids reveled that CZB treatment was responsible for a boost in ALK levels, detected by immunofluorescence (Supplementary Fig. 6C) and western blot (Supplementary Fig. 6D). These data were confirmed at mRNA levels in SW48 and LoVo cells treated with both CZB and ALC (Supplementary Fig. 6E). All together these results suggest that long term ALK inhibition induces in the persistent clones an overall improved differentiation.

### ALK gene signature predicts survival of CMS1 patients

**Fig. 6:**
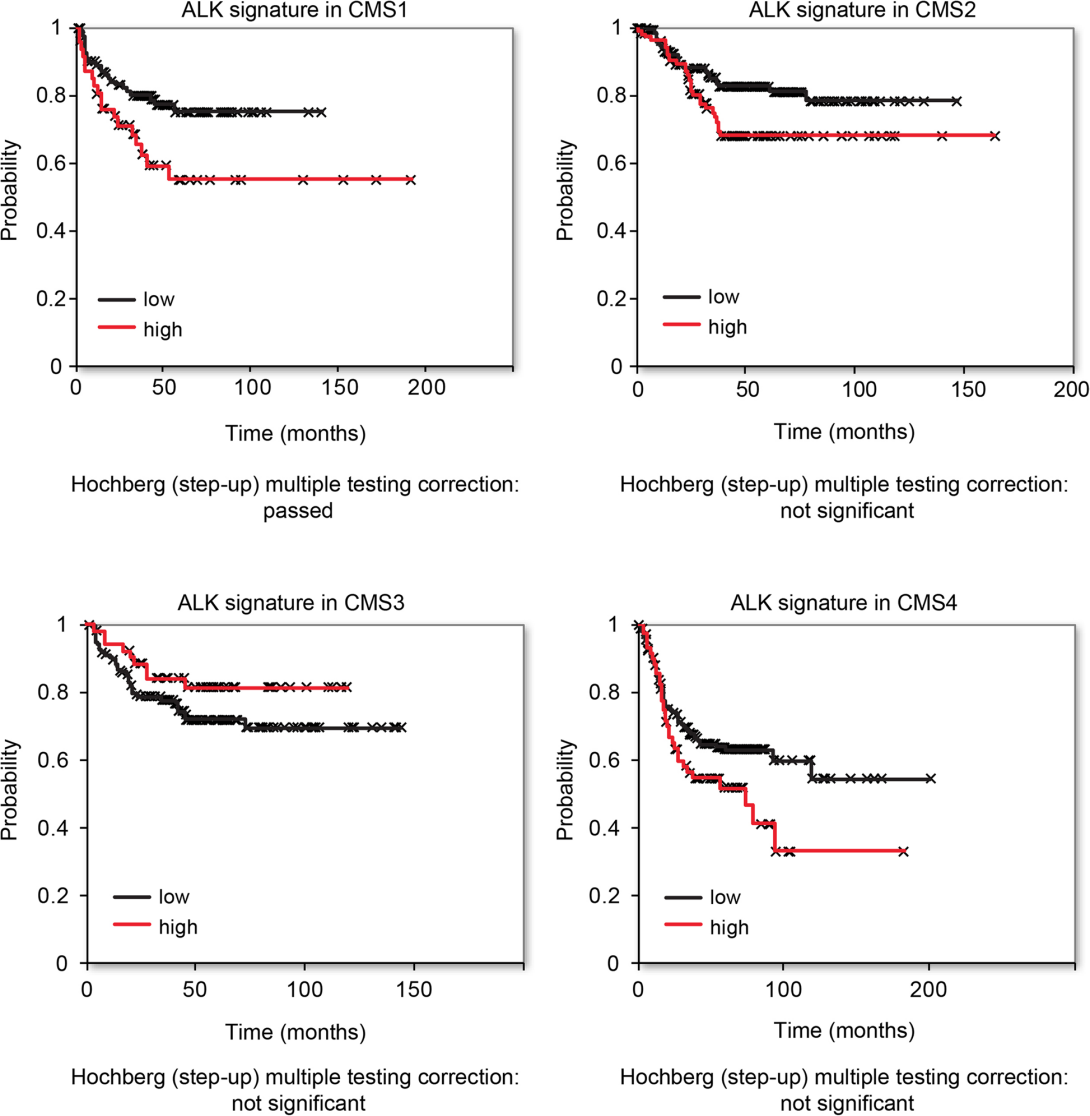
A 65-genes ALK signature is predictive of survival only in CMS1 colon cancer subtype. Bioinformatic study performed on the same dataset of patients employed for the analysis reported in Fig. 1. Subjects were stratified on the basis of CMS classification and high (red line) or low (black line) expression of a signature of 65 ALK-related genes. Data were filtered according to the Hochberg (step-up) multiple testing correction

The inferred ability of ALK expression to predict patient survival prompted us to verify a prognostic role for the ALK-regulated gene signature. Our assumption was that the ALK signaling activation would induce a complex gene expression alteration, which could be tested in the CMSs stratified patient dataset. Because the genomic response to ALK activation is still undefined in CRC and the identification of genes that are specifically transcribed as downstream targets of this receptor remain to be addressed, we referred to a gene expression signature previously reported (GSE41635). In this data set, an unbiased transcriptome analysis was evaluated upon enforced introduction of the full-length ALK wild-type in MCF7 cells. Without filtering, approximately 1000 genes were found to be upregulated with ALK overexpression, all expressed in colon tissue. We set the proportion of false positives (PFPs) to a stringent 5%, in order to obtain a more manageable list of genes (*n* = 65, Supplementary Table 1). The Hochberg (step-up) multiple testing correction was applied and we investigated the comprehensive correlation of this ALK signature with RFS in CRC patients. Strikingly, the ALK signature appears predictive of survival in CMS1, while a very mild (no statistically significant) association of the ALK signature for CMS4 subtype was also detected, but in this case, this subtype failed to pass the Hochberg (step-up) multiple testing correction (Fig. 6).

### ALK inhibition by CZB displays therapeutic potential in patient derived organoids and in mice

**Fig. 7:**
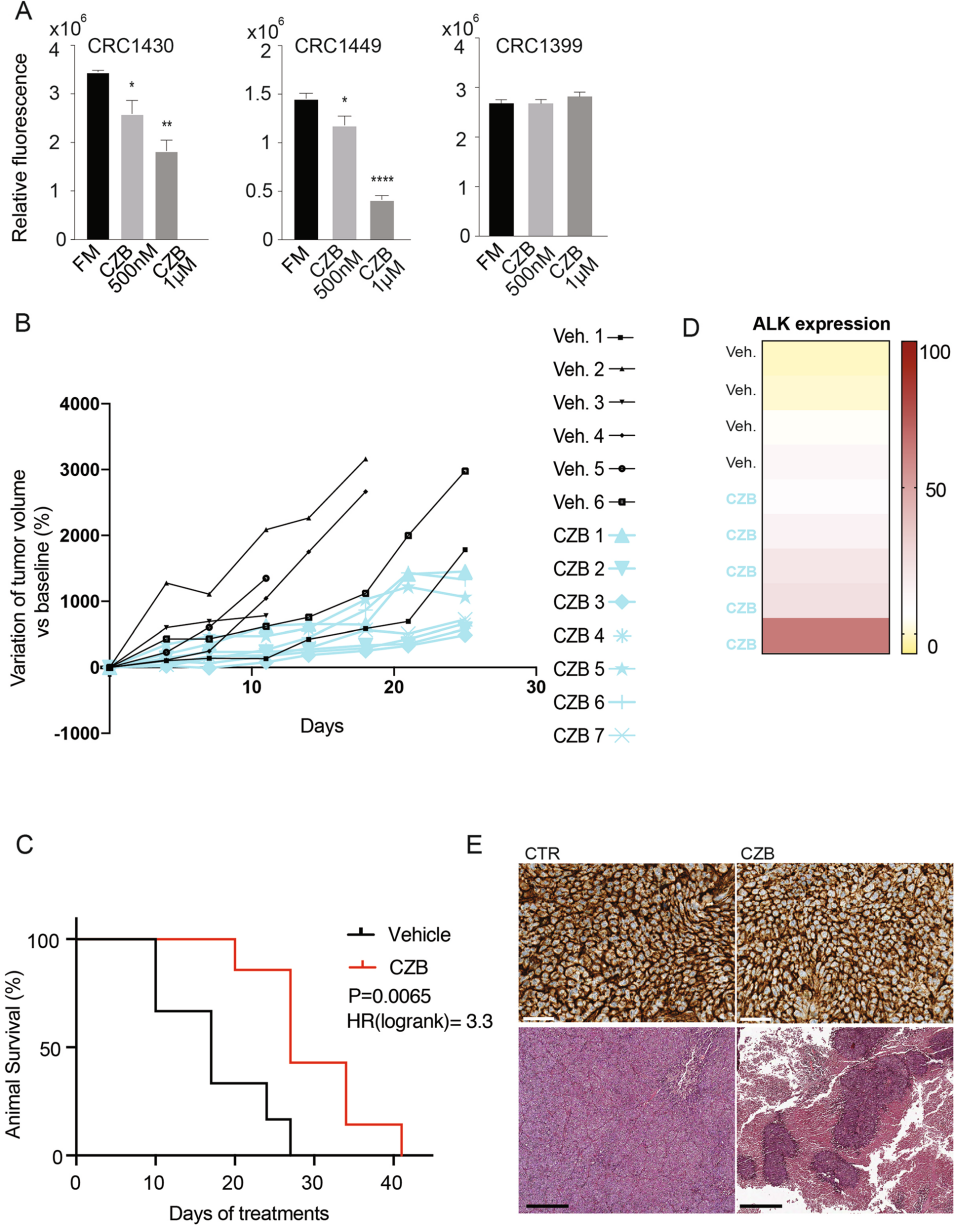
ALK inhibition reduces proliferation of CRC patient-derived organoids in vitro and slows tumour growth in a CMS1 xenograft model. **A** Organoids’ proliferation assessment performed with AlamarBlue after 8/14 days of CZB treatment. Organoids were seeded in 2% BME and CZB treatment was added the following day at the indicated concentrations. Statistics were calculated by one-way ANOVA test. **B** SW48 cells (1 × 10^6^ per animal) were subcutaneously implanted in Nude Foxn1nu mice. Tumor bearing mice were treated with either vehicle control or crizotinib (50 mg/kg/day), by oral gavage. Mice were euthanized when tumor size reached 1.500 mm^3^. Data are means ± SEM from each group (*n* = 7 per treatment group). **C** Survival assessment of Nude Foxn1nu mice from experiment in C. Animal survival percentage is increased in CZB-treated group compared to the control. **D** Heatmap showing ALK mRNA expression measured with qPCR in tumors collected at the endpoint from experiment in C. **E** Histological evaluation of SW48 xenograft tumors extracted from mice by means of ALK abundance (scale bar: 50 μm) and H&E (scale bar: 400 μm)

In order to provide a meaningful translation to the bedside of our findings, we further assessed the involvement of ALK in colorectal patient-derived organoids, by testing the CZB/ALC-sensitivity in this ex-vivo model. CRC1430, CRC1449 and CRC1399 organoids are wild type for clinically validated resistance mutations (*KRAS*, *NRAS*, and *BRAF*) but still not sensitive to EGFR inhibition in vivo. These samples were derived by metastatic lesions of CRC PDXs as previously reported [44]. We found that ALK inhibition induces a growth arrest in CRC1430 and CRC1449 organoids, but has no effect on CRC1399, thus confirming the subtype specificity observed in the cancer cell lines. Both CZB and ALC considerably reduced CRC1430 and CRC1449 proliferation in a dose-dependent manner (Fig. 7A and Supplementary Fig. 3F).

For the assessment of tumor response to ALK inhibition in vivo, we employed immunocompromised nonobese diabetes/severe combined immunodeficiency animals, inoculated with SW48 (CMS1) cells and then randomized into two groups: vehicle control and CZB (50 mg/kg/day). In vivo evaluation showed that CZB strongly affected tumorigenic growth. Indeed, while already after 11 days of treatment in control group tumors reached the limiting size (1500 mm^3^), thus implying the termination of the experiment, the tumor volume average of CZB-treated group was reduced (Fig. 7B), allowing to statistically double the mice survival (*P* = 0.006) (Fig. 7C). A similar experimental setting on 20 more mice, repeated the same results (Supplementary Fig. 7A-B). In addition, as previously reported, no sign of distress or body weight loss was observed in the CZB group (Supplementary Fig. 7C). Next, we tested the ALK mRNA expression levels in tumors collected from the in vivo xenograft experiments. Strikingly, all the CZB treated mice displayed increased amount of ALK mRNA (Fig. 7D), suggesting a positive compensatory feedback enhancing ALK abundance, triggered by ALK inhibition. IHC testing for expression of ALK protein in FFPE tissue sections was performed following the guidelines [45]. Xenograft tumors collected from untreated control mice, revealed a homogeneous macroscopic pattern of ALK protein expression. Nearly, all of the untreated tumors exhibited positive staining for ALK protein, with an intense signal, often with a membrane localization (Fig. 7E). The CZB-treated mice presented a heterogeneous pattern, with some areas weakly positive for ALK staining, suggesting that CZB might impair ALK protein stability or that ALK protein might be regulated differentially within the single tumor cell population. Histologic evaluation of tumors by hematoxylin and eosin staining was performed, including a qualitative assessment of tumor necrosis. A significant increase in tumor focal necrosis severity was seen in tumors from CZB-treated animals compared with vehicle-treated controls. Vehicle-treated animals exhibited only low-grade tumor necrosis, typical of fast-growing malignant tumors, while the treated mice displayed massive necrotic areas, responsible for the impaired growth (Fig. 7E). In conclusion, animal experiment confirmed the in vitro observations, opening important questions on the ALK receptor regulation in CMS1, which will require further evaluations.

## Discussion

The CMS classification has proven to be a useful prognostic factor for early stage colorectal cancer. Deep-learning and large data mining may help to repurpose drugs across all therapeutic areas [46]. By capturing the vast reservoir of information available by preexisting data of tumor gene expression, we unveiled ALK as a new putative driver gene, assessing the impact of its inhibition on CMS outcomes. We infer that particular CRC subtypes show beneficial responses to ALK inhibition, in either adjuvant or metastatic settings. This hypothesis-generating study, stemming from a wide analysis of data obtained from the clinic, showed that ALK mRNA abundance, as well as ALK pathway activation are associated with survival in CMS1 patients, while displaying no effect in the other CMSs. Notably, evidence for a possible involvement of ALK in colorectal cancer has already been suggested, mostly arguing for a cross-talk with EGFR and revealing the presence of tumors harboring both tyrosine kinase receptors alteration or ALK gene amplification as a poor prognostic factor [26]. Our data on the in vitro sensitivity to ALK inhibition were congruent in multiple cell lines and settings, from proliferation to soft agar and three-dimensional assays, which are known to be closer to tumors in vivo. The cellular systems we applied reliably recapitulate the CMS1 and strongly support the therapeutic inhibition of ALK in this specific CRC subset, while six additional cell lines belonging to CMS2/3 and 4 confirmed the complete lack of response. Upon validation by a wide array of in vitro studies, we could confirm the findings by employing an in vivo model, which exhibited animal-specific variation in terms of onset and rate of growth, under CZB treatment. Mechanistically, ALK mediated cancer growth inhibition depended on the ALK mRNAs copy number, therefore these results call for a careful evaluation of the ALK full-length abundance in CRC specimen, to schedule the right treatment. The efficacy of ALK inhibition is responsible for the impairment of the AKT axis, while almost no effect was detected on the ERK activation. On the other hand, the same involvement of AKT axis was observed after ALK stimulation with its cognate ligands, particularly with ALKAL2.

Stemming from these observations, we propose a model in which an autocrine loop of ALKAL2-mediated ALK activation, by signaling through the AKT downstream cascade, is responsible for the CMS1 cancer progression. Of note, these findings are strikingly in line with a recent report claiming that the sensitivity to ALK TKI is mediated by the amount of ALKAL2 ligand [47].

Finally, a comprehensive imaging analysis of the 3D tumor growth, revealed a functional role for ALK in the formation of the tumor bulk in CMS1. From a clinical point of view, metastatic CRC maintains a unique dependency on the receptors of the ErbB family, which are the target of few monoclonal antibodies that robustly downregulate EGFR signaling. Nevertheless, the emergence of drug resistance limits the efficacy of these therapies. We are aware that experiments with cell lines cannot recapitulate the wide complexity of tumors described on a population basis. Nevertheless, here the cell line work was tightly supported and in line with the patient-derived data and Kaplan-Meier profiles. The observation that *ALK* is relevant in a small percentage of CRC patient is in agreement with previous reports generated on unselected colorectal cancers specimens [25, 26]. The unexpected finding here is the identification of an ALKAL2-mediated autocrine loop activating the ALK axis specifically in the CMS1, with consequent therapeutic implications for this patient group. Notably, ALK translocation is usually associated to genomic instability [48], which is also a feature of the CMS1 subtype. Moreover, in our work, preclinical use of ALK inhibition as therapeutic strategy was validated by the exploitation of in vivo mice models. Interestingly, these data might find support in some clinical activity registered for the entrectinib [49], in patients with *ALK* translocation in gastrointestinal cancer, who had never been treated with agents designed to target those alterations.

## Conclusions

This work highlights the growing assumption that integration of clinical data with functional analyses of preclinical models can lead to rational therapies aimed at delaying or halting the occurrence of CRC in specific CMSs. To sum up, here we suggest that ALK pathway may represent a target for personalized therapy in CRC patients belonging to the CMS1. These results call for the development of ALK neutralizing antibodies, in order to potentiate the poor armamentarium of drugs available for CRC patients. Indeed, the employment of ALK neutralizing antibody rather than TKI may represent a successful strategy to block the ALKAL2/ALK autocrine loop driving cancer growth in patients that currently have very limited treatment options.

## Supplementary Information

**Additional file 1.** Supplementary Information.

**Additional file 2: Supplementary Figure 1**. ALK status in a panel of CRC cell lines belonging to different consensus molecular subtypes.

**Additional file 3: Supplementary Figure 2**. ALK inhibition significantly impacts on CMS1 spheroids growth.

**Additional file 4: Supplementary Figure 3**. ALK inhibitor alectinib reduces proliferation of CMS1 cells in 2D and 3D settings and in a model of CRC patient-derived organoid.

**Additional file 5: Supplementary Figure 4**. ALK inhibition triggers apoptosis in CMS1 cell lines.

**Additional file 6: Supplementary Figure 5**. ALK and its downstream pathway evaluation in colorectal cancer cell lines.

**Additional file 7: Supplementary Figure 6**. ALK is highly expressed and activated in CMS1 cells and CZB boosts ALK protein expression.

**Additional file 8: Supplementary Figure 7**. CZB displays limited toxicity in mice xenografts.

**Additional file 9: Table S1**.

**Additional file 10.**

**Additional file 11.**

**Additional file 12.**

**Additional file 13.**

**Additional file 14.**

**Additional file 15.**

**Additional file 16.**

**Additional file 17.**

## Data Availability

The datasets analysed during the current study are available in the KM plot repository and gene expression data and relapse free and overall survival information are downloaded from GEO, EGA and TCGA. [https://kmplot.com/analysis/].

## Abbreviations

ALK: Anaplastic lymphoma kinase
CRC: Colorectal cancer
CMS: Consensus molecular subtype
IHC: Immunohistochemistry
CZB: Crizotinib
ALC: Alectinib
TKI: Tyrosine kinase inhibitor
EMT: Epithelial Mesenchymal Transition
ALKAL2: ALK and LTK ligand 2.
