# Supplementary material for: The autocrine loop of ALK receptor and ALKAL2 ligand is an actionable target in consensus molecular subtype 1 colon cancer
Source: J Exp Clin Cancer Res. 2022 Mar 29;41:113. doi: 10.1186/s13046-022-02309-1 (PMC8962179; doi:10.1186/s13046-022-02309-1)
Supplement: Supplementary file 1 — Additional file 1. Supplementary Information. [file 13046_2022_2309_MOESM1_ESM.docx]

**LIST OF REAGENTS**

ANTIBODIES LIST

| ANTIBODY | SOURCE | CATALOG CODE |
| --- | --- | --- |
| Phospho-AKT (Ser473) (D9E) XP Rabbit monoclonal antibody | Cell Signalling Technology | #4060 |
| AKT Rabbit polyclonal antibody | Cell Signalling Technology | #9272 |
| MAP Kinase activated (diphosphorylated ERK 1/2) Mouse monoclonal antibody | Sigma-Aldrich | M8159 |
| ERK 2 (D-2) Mouse monoclonal antibody | Santa Cruz Biotechnology | sc-1647 |
| ALK (F-12) Mouse monoclonal antibody | Santa Cruz Biotechnology | sc-1647 |
| ALK (D5F3) XP Rabbit mAb | Cell signalling Technologies | #3633 |
| pALK (Tyr1604) Rabbit monoclonal antibody | Cell signalling Technologies | #3341 |
| β-Actin (C4) Mouse monoclonal antibody | Santa Cruz Biotechnology | sc-47778 |
| α-Tubulin (B-7) Mouse monoclonal antibody | Santa Cruz Biotechnology | sc-5286 |
| E-cadherin (G-10) Mouse monoclonal antibody | Santa Cruz Biotechnology | sc-8426 |
| Anti-Mouse/Anti-Rabbit EnVision+ System- HRP Labelled Polymer | Dako | K4001/K4003 |
| Anti-BrdU | DSHB | #G3G4  (AB_2618097) |
| Cy™3 AffiniPure Goat Anti-Rabbit IgG (H+L) | Jackson ImmunoResearch | 111-165-003 |
| Alexa Fluor® 488-AffiniPure Goat Anti-Rabbit IgG (H+L) | Jackson ImmunoResearch | 111-545-003 |
| Anti-ALK D5F3 rabbit monoclonal antibody | Ventana Medical Systems (Roche) | 790-4796 |
| Anti-Vimentin V9 mouse antibody | Ventana Medical Systems (Roche) | 790-2917 |
| Anti-E-cadherin (36) mouse monoclonal antibody | Ventana Medical Systems (Roche) | 790-4497 |

FLUORESCENT PROBES LIST

| FLUORESCENT PROBE | SOURCE | CATALOG CODE |
| --- | --- | --- |
| Phalloidin-TRITC | Sigma-Aldrich | P1951 |
| DAPI | Sigma-Aldrich | #D9542 |
| IncuCyte® Caspase-3/7 Green Apoptosis Assay Reagent | Essen BioScience Inc | Cat. No. 4440 |
| IncuCyte® Cytotox Green Reagent for Counting Dead Cells | Essen BioScience Inc | Cat. No. 4633 |

PRIMERS FOR qRT-PCR LIST

| TARGET TRANSCRIPT | SEQUENCE | SOURCE |
| --- | --- | --- |
| BCL2 | FW: ATGTGTGTGGAGAGCGTCAACC | Sigma-Aldrich |
|  | RW: TGAGCAGAGTCTTCAGAGACAGC | Sigma-Aldrich |
| BAX | FW: GGGACGAACTGGACAGTAACA | Sigma-Aldrich |
|  | RW: CCGCCACAAAGATGGTCA | Sigma-Aldrich |
| VIM | FW: GGAAACTAATCTGGATTCACTC | Sigma-Aldrich |
|  | RW: CATCTCTAGTTTCAACCGTC | Sigma-Aldrich |
| B2M | FW: TGCCTGCCGTGTGAACCATGT | Sigma-Aldrich |
|  | RW: TGCGGCATCTTCAAACCTCCATGA | Sigma-Aldrich |
| ALK | FW: AATACAGCACCCAAATCAAG | Sigma-Aldrich |
|  | RW: GAACTGTAGGGTCAAAGATG | Sigma-Aldrich |
| ALKAL1 | FW: CACCGACTCTATTACAATACC | Sigma-Aldrich |
|  | RW: GGAAATGTAGGGTAGTTTTGC | Sigma-Aldrich |
| ALKAL2 | FW: ACAGACTCTTCATAAGTCC | Sigma-Aldrich |
|  | RW: TGCGATTTCACCTAATGAAG | Sigma-Aldrich |

**SUPPLEMENTARY METHODS**

SOFT AGAR ASSAY

Cells were grown embedded into a layer of sterile agar 0.3% in full medium plus proper treatments, on wells previously coated with agar 0.6% as previously described for spheroid assays. A thin growth medium layer was added weekly on top of agar layer to prevent desiccation. After 15 days-1 month, depending on each cell line, photos of 3D colonies were taken using inverted microscope (Leitz Labovert FS, code 515). Embedded cells were then incubated with PFA4% for fixation and stained with Giemsa 3-5% in PBS 1X. Photographs of each well were taken and colonies’ number was assessed with ImageJ Cell Counter plugin.

FLUORESCENT IN SITU HYBRIDIZATION

Samples consisted of small formalin-fixed and paraffin-embedded cell pellets. FISH assay was performed using the Vysis ALK Break Apart FISH Probe Kit (Abbott). This break-apart FISH test is based on a mixture of two probes hybridizing to the proximal (3’, orange-labeled probe) and distal (5’, green-labeled probe) to the ALK breakpoint cluster region. At least 50 non-overlapping nuclei were scored for each specimen by a trained technologist and a pathologist. Cells positive for rearrangement are defined by two main patterns: I) a “split pattern”, with 3’ and 5’ break apart signals at a distance of two times the diameter of the largest signal; II) a “5’ deletion pattern”, showing one fusion signal and an isolated 3’ orange signal (without the corresponding 5’ green signal). A case was considered FISH positive for ALK rearrangements when at least 15% of tumour cells showed any split or any 5’ deletion pattern.

IMMUNOFLUORESCENCE IN 3D SETTINGS

Cells grown in low-attachment conditions as described in spheroid assay method section were collected, formalin-fixed and paraffin-embedded (FFPE). Specimens were sectioned, and slides were deparaffinized with xylene. After performing antigen retrieval using the EDTA buffer, samples were permeabilized, blocked with 5% BSA, 0,1% Triton in PBS, and incubated O/N at 4°C with ALK D5F3 primary antibody. The day after, slides were stained with anti-rabbit secondary antibody (Cy3) and DAPI. Images detection was performed with Olympus VS200 slide scanner.

FLOW CYTOMETRY ANALYSES OF SURFACE RECEPTORS

To evaluate ALK surface receptor levels, cells were treated with harvested and fixed in PFA4% for 15’ at RT. Cells were then washed once in PBS and incubated with ALK D5F3 antibody for 1h at RT. After washing in PBS, cells were incubated with green secondary antibody for 30’ at RT. Fluorescence intensity was measured using Cytoflex S Flow Cytometer and data were analyzed by CytExpert software.
