# Supplementary figures and images for: The autocrine loop of ALK receptor and ALKAL2 ligand is an actionable target in consensus molecular subtype 1 colon cancer
Source: J Exp Clin Cancer Res. 2022 Mar 29;41:113. doi: 10.1186/s13046-022-02309-1 (PMC8962179; doi:10.1186/s13046-022-02309-1)

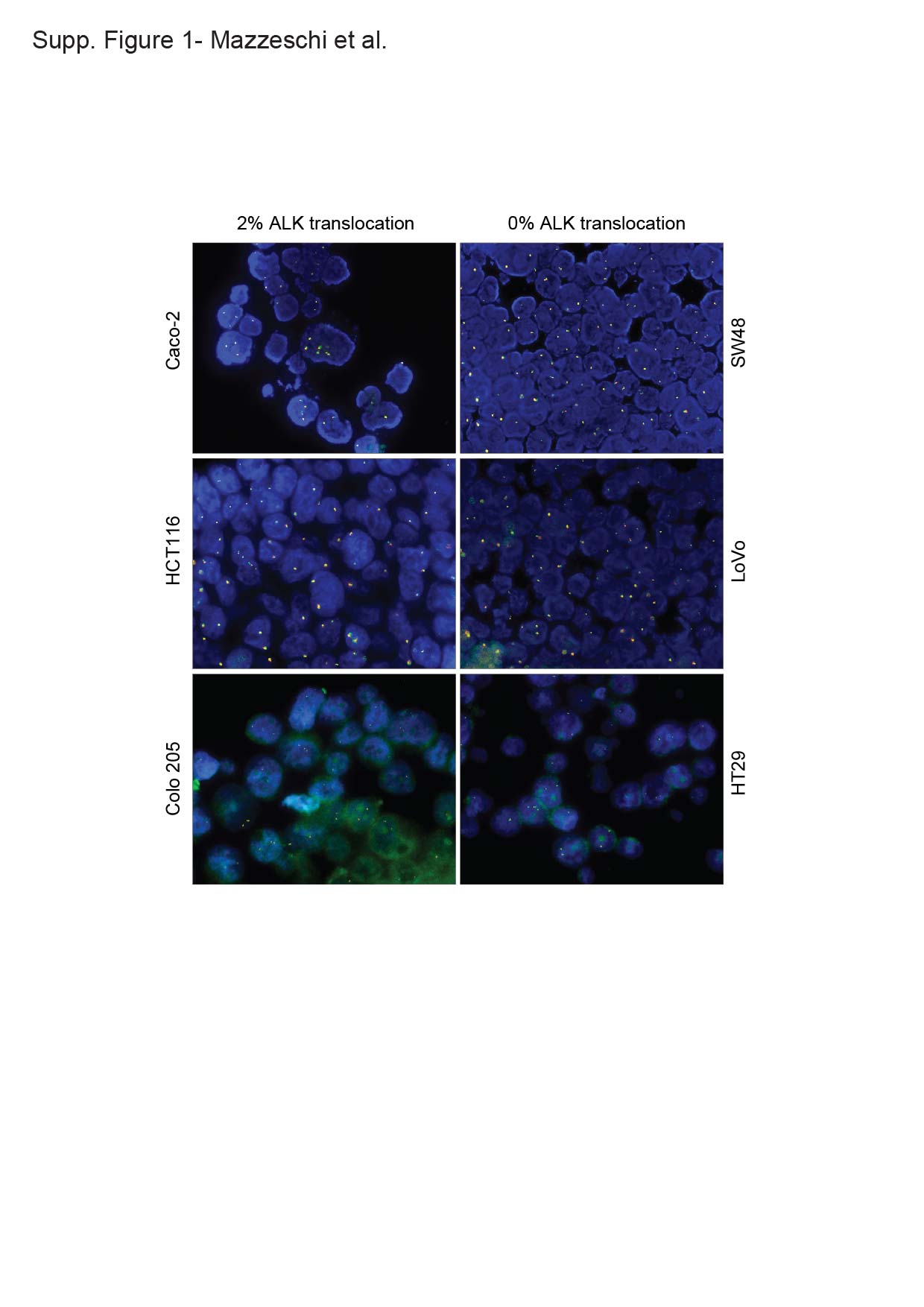

Supplement: Supplementary file 2 — Additional file 2: Supplementary Figure 1. ALK status in a panel of CRC cell lines belonging to different consensus molecular subtypes. [file 13046_2022_2309_MOESM2_ESM.jpg]

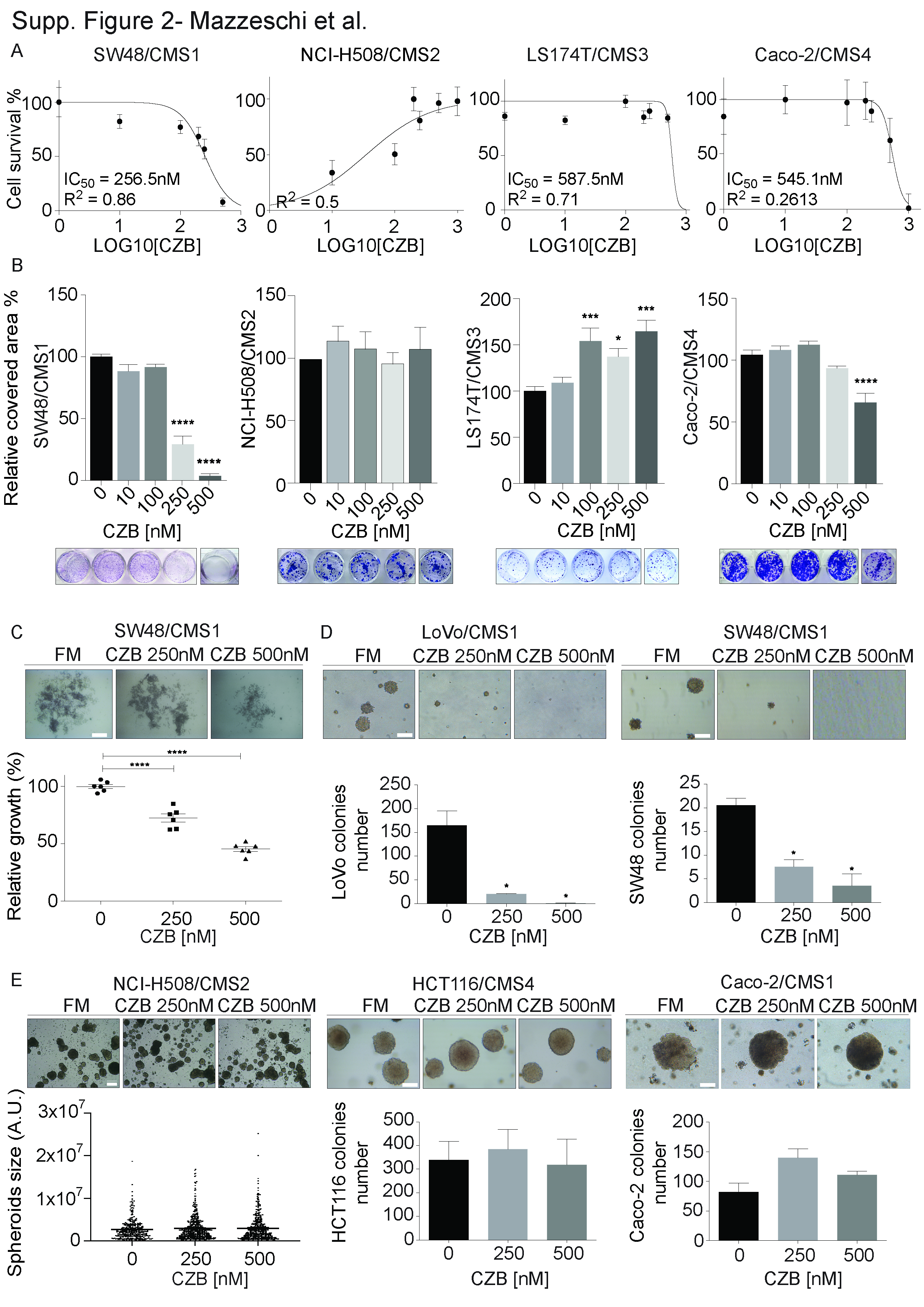

Supplement: Supplementary file 3 — Additional file 3: Supplementary Figure 2. ALK inhibition significantly impacts on CMS1 spheroids growth. [file 13046_2022_2309_MOESM3_ESM.jpg]

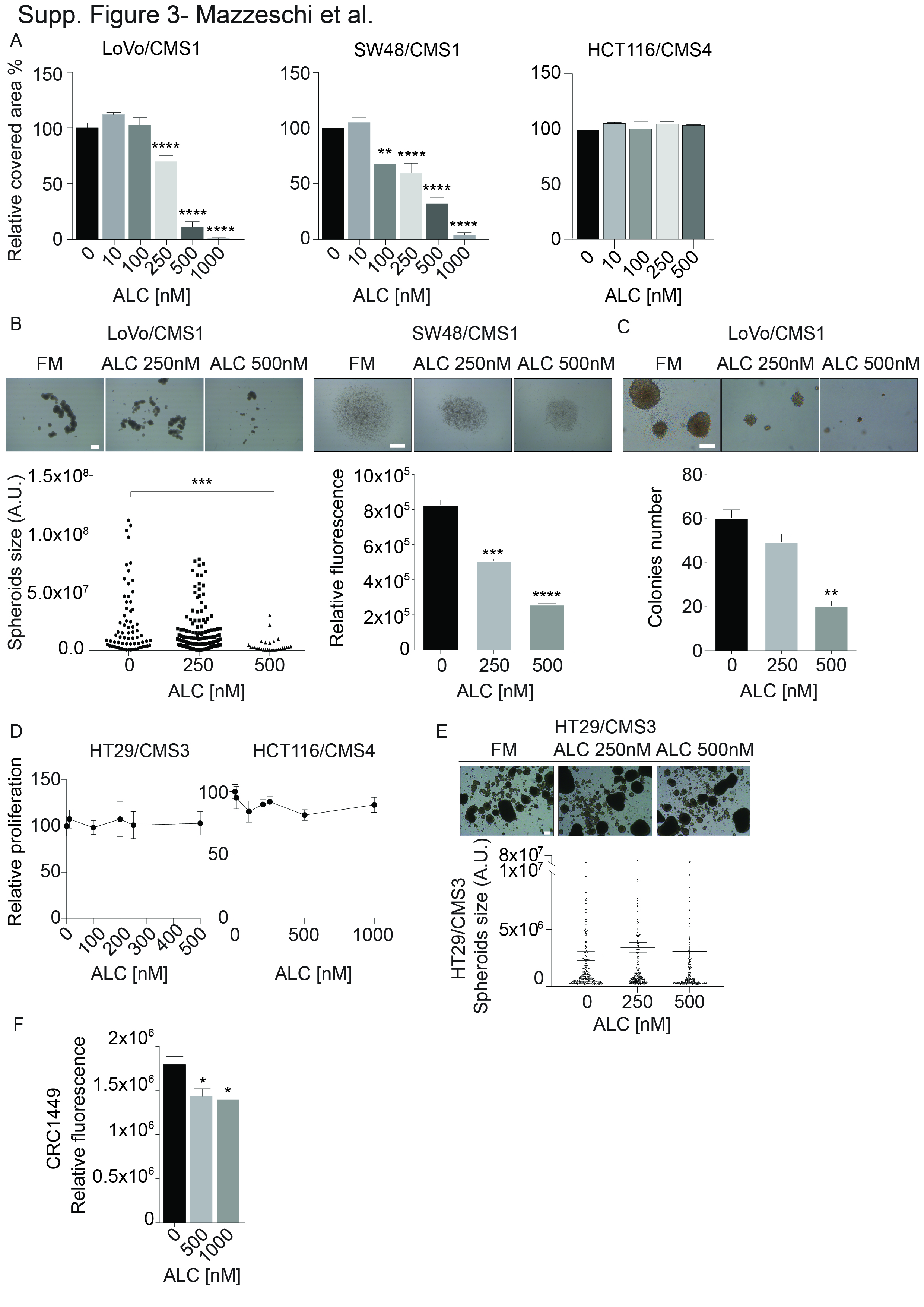

Supplement: Supplementary file 4 — Additional file 4: Supplementary Figure 3. ALK inhibitor alectinib reduces proliferation of CMS1 cells in 2D and 3D settings and in a model of CRC patient-derived organoid. [file 13046_2022_2309_MOESM4_ESM.jpg]

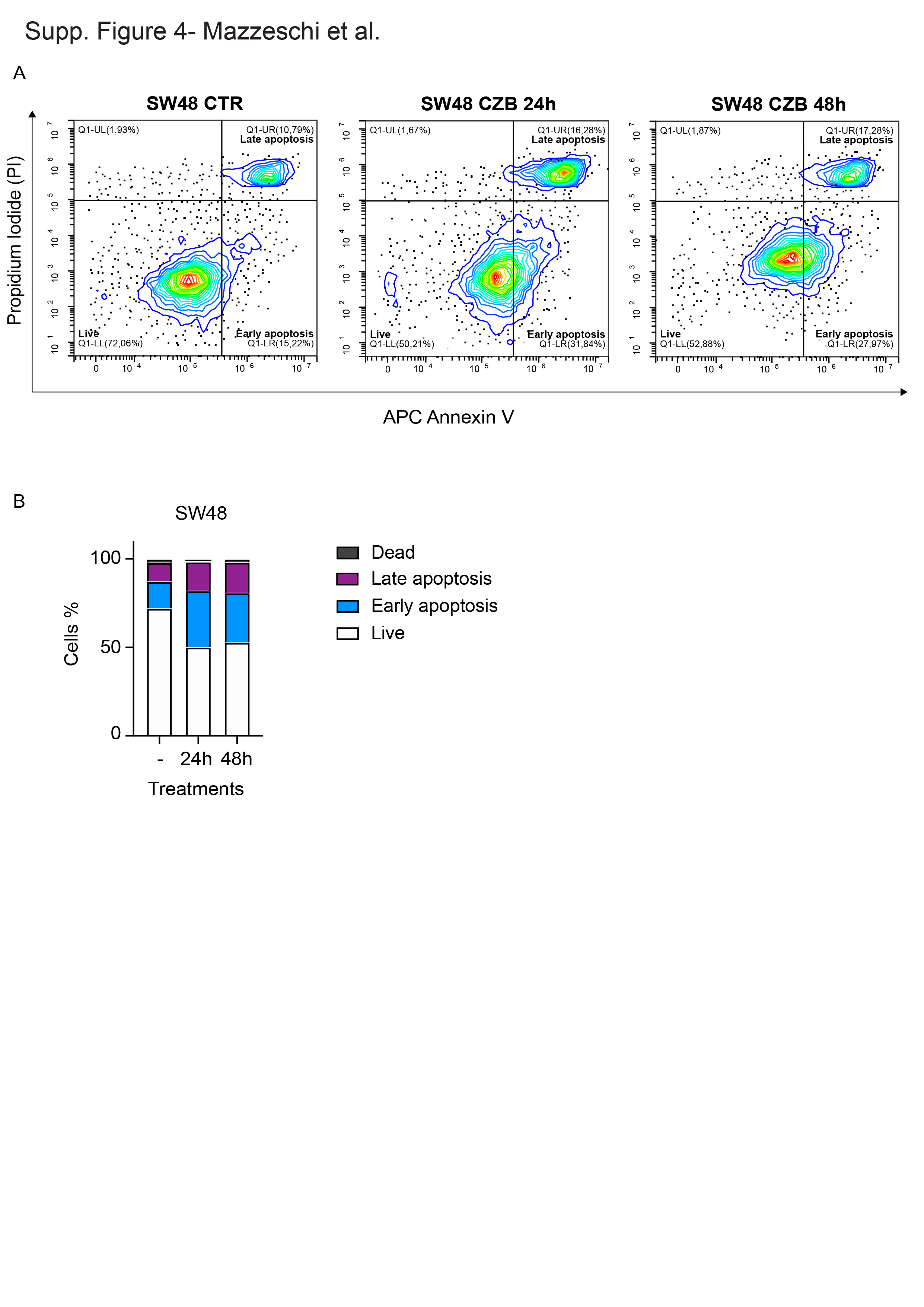

Supplement: Supplementary file 5 — Additional file 5: Supplementary Figure 4. ALK inhibition triggers apoptosis in CMS1 cell lines. [file 13046_2022_2309_MOESM5_ESM.jpg]

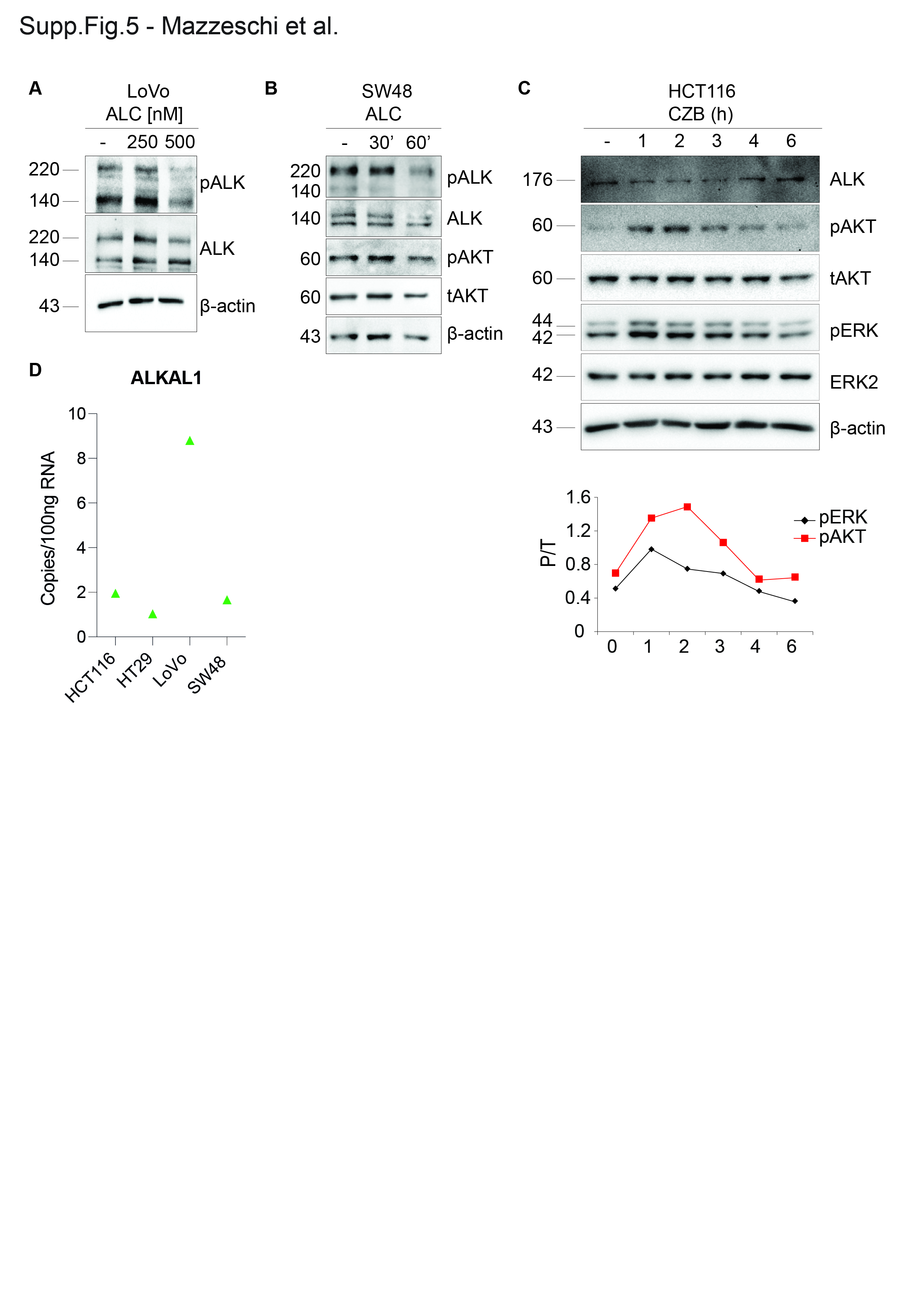

Supplement: Supplementary file 6 — Additional file 6: Supplementary Figure 5. ALK and its downstream pathway evaluation in colorectal cancer cell lines. [file 13046_2022_2309_MOESM6_ESM.jpg]

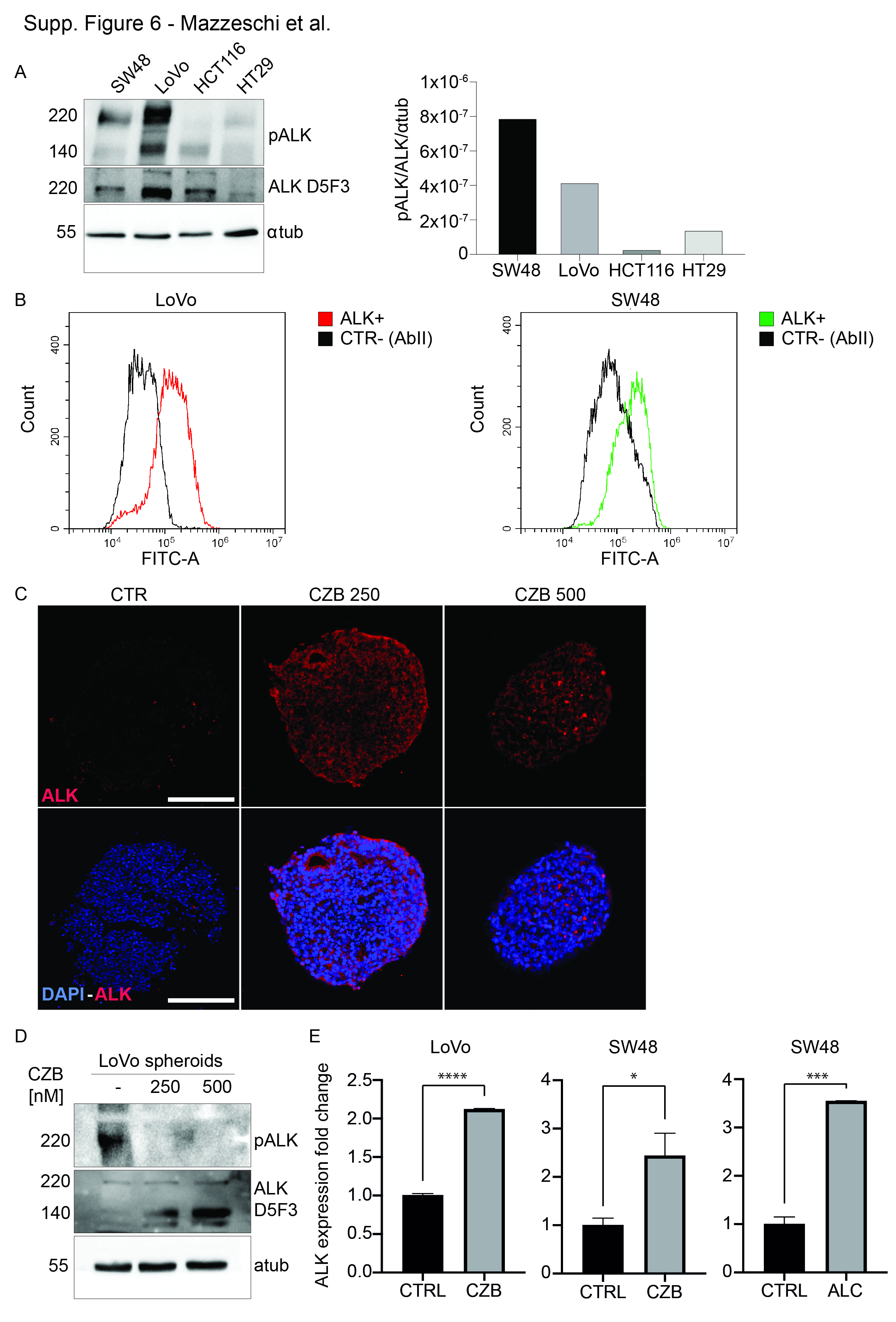

Supplement: Supplementary file 7 — Additional file 7: Supplementary Figure 6. ALK is highly expressed and activated in CMS1 cells and CZB boosts ALK protein expression. [file 13046_2022_2309_MOESM7_ESM.jpg]

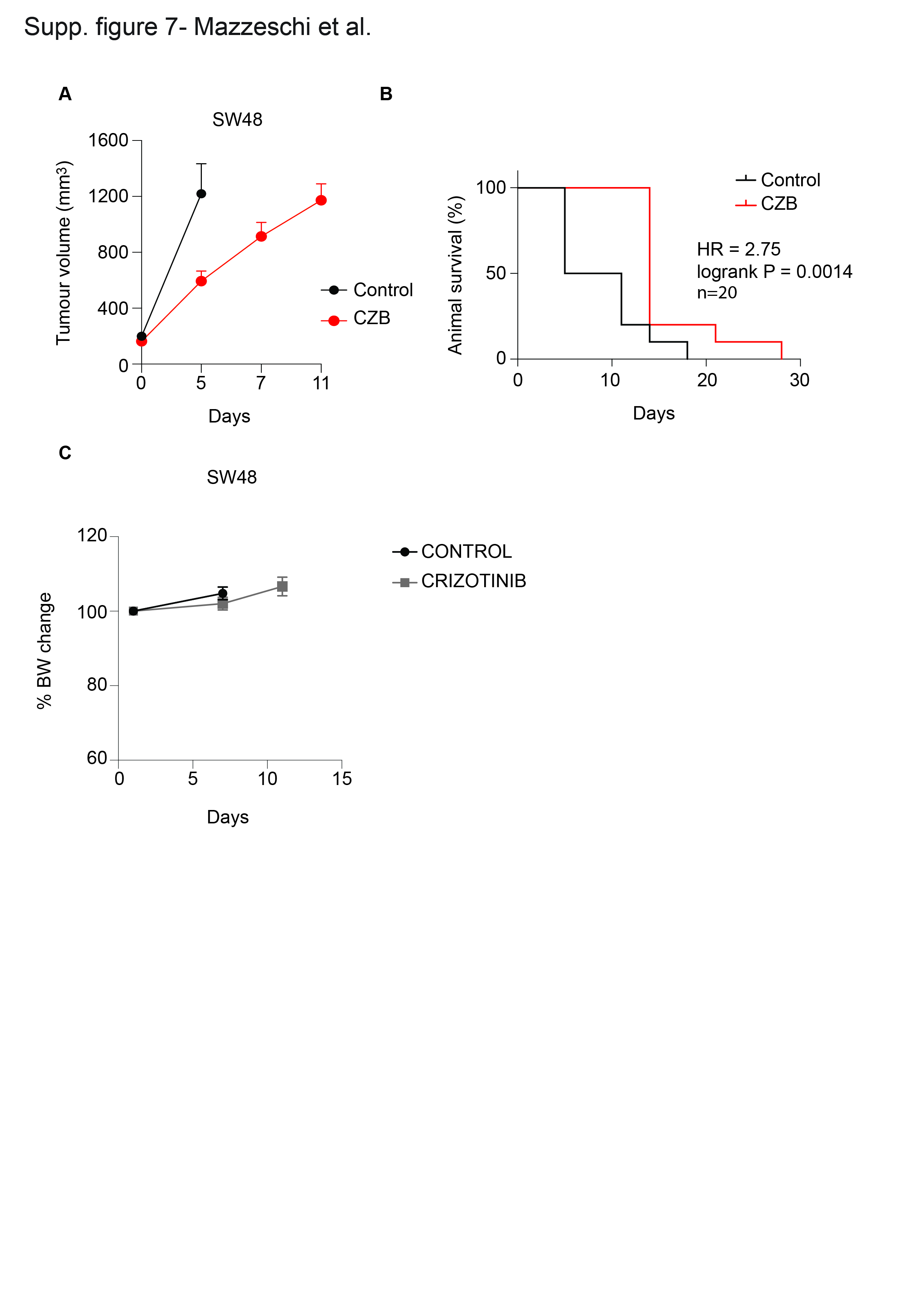

Supplement: Supplementary file 8 — Additional file 8: Supplementary Figure 7. CZB displays limited toxicity in mice xenografts. [file 13046_2022_2309_MOESM8_ESM.jpg]
